# Polygenic risk modeling for prediction of epithelial ovarian cancer risk

**DOI:** 10.1038/s41431-021-00987-7

**Published:** 2022-01-14

**Authors:** Eileen O. Dareng, Jonathan P. Tyrer, Daniel R. Barnes, Michelle R. Jones, Xin Yang, Katja K. H. Aben, Muriel A. Adank, Simona Agata, Irene L. Andrulis, Hoda Anton-Culver, Natalia N. Antonenkova, Gerasimos Aravantinos, Banu K. Arun, Annelie Augustinsson, Judith Balmaña, Elisa V. Bandera, Rosa B. Barkardottir, Daniel Barrowdale, Matthias W. Beckmann, Alicia Beeghly-Fadiel, Javier Benitez, Marina Bermisheva, Marcus Q. Bernardini, Line Bjorge, Amanda Black, Natalia V. Bogdanova, Bernardo Bonanni, Ake Borg, James D. Brenton, Agnieszka Budzilowska, Ralf Butzow, Saundra S. Buys, Hui Cai, Maria A. Caligo, Ian Campbell, Rikki Cannioto, Hayley Cassingham, Jenny Chang-Claude, Stephen J. Chanock, Kexin Chen, Yoke-Eng Chiew, Wendy K. Chung, Kathleen B. M. Claes, Sarah Colonna, GEMO Study Collaborators, Fabienne Lesueur, Noura Mebirouk, GC-HBOC Study Collaborators, Christoph Engel, Rita K. Schmutzler, EMBRACE Collaborators, Daniel Barrowdale, Eleanor Davies, Diana M. Eccles, D. Gareth Evans, Linda S. Cook, Fergus J. Couch, Mary B. Daly, Fanny Dao, Eleanor Davies, Miguel de la Hoya, Robin de Putter, Joe Dennis, Allison DePersia, Peter Devilee, Orland Diez, Yuan Chun Ding, Jennifer A. Doherty, Susan M. Domchek, Thilo Dörk, Andreas du Bois, Matthias Dürst, Diana M. Eccles, Heather A. Eliassen, Christoph Engel, Gareth D. Evans, Peter A. Fasching, James M. Flanagan, Renée T. Fortner, Eva Machackova, Eitan Friedman, Patricia A. Ganz, Judy Garber, Francesca Gensini, Graham G. Giles, Gord Glendon, Andrew K. Godwin, Marc T. Goodman, Mark H. Greene, Jacek Gronwald, OPAL Study Group, AOCS Group, Eric Hahnen, Christopher A. Haiman, Niclas Håkansson, Ute Hamann, Thomas V. O. Hansen, Holly R. Harris, Mikael Hartman, Florian Heitz, Michelle A. T. Hildebrandt, Estrid Høgdall, Claus K. Høgdall, John L. Hopper, Ruea-Yea Huang, Chad Huff, Peter J. Hulick, David G. Huntsman, Evgeny N. Imyanitov, KConFab Investigators, Georgia Chenevix-Trench, HEBON Investigators, Muriel A. Adank, Peter Devilee, Annemieke H. van der Hout, Claudine Isaacs, Anna Jakubowska, Paul A. James, Ramunas Janavicius, Allan Jensen, Oskar Th. Johannsson, Esther M. John, Michael E. Jones, Daehee Kang, Beth Y. Karlan, Anthony Karnezis, Linda E. Kelemen, Elza Khusnutdinova, Lambertus A. Kiemeney, Byoung-Gie Kim, Susanne K. Kjaer, Ian Komenaka, Jolanta Kupryjanczyk, Allison W. Kurian, Ava Kwong, Diether Lambrechts, Melissa C. Larson, Conxi Lazaro, Nhu D. Le, Goska Leslie, Jenny Lester, Fabienne Lesueur, Douglas A. Levine, Lian Li, Jingmei Li, Jennifer T. Loud, Karen H. Lu, Jan Lubiński, Phuong L. Mai, Siranoush Manoukian, Jeffrey R. Marks, Rayna Kim Matsuno, Keitaro Matsuo, Taymaa May, Lesley McGuffog, John R. McLaughlin, Iain A. McNeish, Noura Mebirouk, Usha Menon, Austin Miller, Roger L. Milne, Albina Minlikeeva, Francesmary Modugno, Marco Montagna, Kirsten B. Moysich, Elizabeth Munro, Katherine L. Nathanson, Susan L. Neuhausen, Heli Nevanlinna, Joanne Ngeow Yuen Yie, Henriette Roed Nielsen, Finn C. Nielsen, Liene Nikitina-Zake, Kunle Odunsi, Kenneth Offit, Edith Olah, Siel Olbrecht, Olufunmilayo I. Olopade, Sara H. Olson, Håkan Olsson, Ana Osorio, Laura Papi, Sue K. Park, Michael T. Parsons, Harsha Pathak, Inge Sokilde Pedersen, Ana Peixoto, Tanja Pejovic, Pedro Perez-Segura, Jennifer B. Permuth, Beth Peshkin, Paolo Peterlongo, Anna Piskorz, Darya Prokofyeva, Paolo Radice, Johanna Rantala, Marjorie J. Riggan, Harvey A. Risch, Cristina Rodriguez-Antona, Eric Ross, Mary Anne Rossing, Ingo Runnebaum, Dale P. Sandler, Marta Santamariña, Penny Soucy, Rita K. Schmutzler, V. Wendy Setiawan, Kang Shan, Weiva Sieh, Jacques Simard, Christian F. Singer, Anna P. Sokolenko, Honglin Song, Melissa C. Southey, Helen Steed, Dominique Stoppa-Lyonnet, Rebecca Sutphen, Anthony J. Swerdlow, Yen Yen Tan, Manuel R. Teixeira, Soo Hwang Teo, Kathryn L. Terry, Mary Beth Terry, The OCAC Consortium, Eileen O. Dareng, Jonathan P. Tyrer, Michelle R. Jones, Katja K. H. Aben, Hoda Anton-Culver, Natalia N. Antonenkova, Gerasimos Aravantinos, Matthias W. Beckmann, Alicia Beeghly-Fadiel, Javier Benitez, Marina Bermisheva, Marcus Q. Bernardini, Line Bjorge, Natalia V. Bogdanova, James D. Brenton, Agnieszka Budzilowska, Ralf Butzow, Hui Cai, Ian Campbell, Rikki Cannioto, Jenny Chang-Claude, Stephen J. Chanock, Kexin Chen, Yoke-Eng Chiew, Linda S. Cook, Fanny Dao, Joe Dennis, Jennifer A. Doherty, Thilo Dörk, Andreas du Bois, Matthias Dürst, Diana M. Eccles, Heather A. Eliassen, Peter A. Fasching, James M. Flanagan, Renée T. Fortner, Graham G. Giles, Marc T. Goodman, Jacek Gronwald, Christopher A. Haiman, Niclas Håkansson, Holly R. Harris, Florian Heitz, Michelle A. T. Hildebrandt, Estrid Høgdall, Claus K. Høgdall, Ruea-Yea Huang, Chad Huff, David G. Huntsman, Anna Jakubowska, Allan Jensen, Michael E. Jones, Daehee Kang, Beth Y. Karlan, Anthony Karnezis, Linda E. Kelemen, Elza Khusnutdinova, Lambertus A. Kiemeney, Byoung-Gie Kim, Susanne K. Kjaer, Jolanta Kupryjanczyk, Diether Lambrechts, Melissa C. Larson, Nhu D. Le, Jenny Lester, Douglas A. Levine, Karen H. Lu, Jan Lubiński, Jeffrey R. Marks, Rayna Kim Matsuno, Keitaro Matsuo, Taymaa May, John R. McLaughlin, Iain A. McNeish, Roger L. Milne, Albina Minlikeeva, Francesmary Modugno, Kirsten B. Moysich, Elizabeth Munro, Heli Nevanlinna, Kunle Odunsi, Siel Olbrecht, Sara H. Olson, Håkan Olsson, Ana Osorio, Sue K. Park, Tanja Pejovic, Jennifer B. Permuth, Anna Piskorz, Darya Prokofyeva, Marjorie J. Riggan, Harvey A. Risch, Cristina Rodriguez-Antona, Mary Anne Rossing, Ingo Runnebaum, Dale P. Sandler, V. Wendy Setiawan, Kang Shan, Weiva Sieh, Honglin Song, Melissa C. Southey, Helen Steed, Rebecca Sutphen, Anthony J. Swerdlow, Soo Hwang Teo, Kathryn L. Terry, Pamela J. Thompson, Liv Cecilie Vestrheim Thomsen, Linda Titus, Britton Trabert, Ruth Travis, Shelley S. Tworoger, Ellen Valen, Anne M. van Altena, Els Van Nieuwenhuysen, Digna Velez Edwards, Robert A. Vierkant, Frances Wang, Penelope M. Webb, Clarice R. Weinberg, Nicolas Wentzensen, Emily White, Alice S. Whittemore, Stacey J. Winham, Alicja Wolk, Yin-Ling Woo, Anna H. Wu, Li Yan, Drakoulis Yannoukakos, Wei Zheng, Argyrios Ziogas, Kate Lawrenson, Anna deFazio, Susan J. Ramus, Celeste L. Pearce, Alvaro N. Monteiro, Julie M. Cunningham, Ellen L. Goode, Joellen M. Schildkraut, Andrew Berchuck, Simon A. Gayther, Paul D. P. Pharoah, The CIMBA Consortium, Daniel R. Barnes, Xin Yang, Muriel A. Adank, Simona Agata, Irene L. Andrulis, Banu K. Arun, Annelie Augustinsson, Judith Balmaña, Rosa B. Barkardottir, Daniel Barrowdale, Bernardo Bonanni, Ake Borg, Saundra S. Buys, Maria A. Caligo, Hayley Cassingham, Wendy K. Chung, Kathleen B. M. Claes, Sarah Colonna, Fergus J. Couch, Mary B. Daly, Eleanor Davies, Miguel de la Hoya, Robin de Putter, Allison DePersia, Peter Devilee, Orland Diez, Yuan Chun Ding, Susan M. Domchek, Diana M. Eccles, Christoph Engel, D. Gareth Evans, Eva Machackova, Eitan Friedman, Patricia A. Ganz, Judy Garber, Francesca Gensini, Gord Glendon, Andrew K. Godwin, Mark H. Greene, Eric Hahnen, Ute Hamann, Thomas V. O. Hansen, Mikael Hartman, John L. Hopper, Peter J. Hulick, Evgeny N. Imyanitov, Claudine Isaacs, Paul A. James, Ramunas Janavicius, Oskar Th. Johannsson, Esther M. John, Ian Komenaka, Allison W. Kurian, Ava Kwong, Conxi Lazaro, Goska Leslie, Fabienne Lesueur, Jingmei Li, Jennifer T. Loud, Phuong L. Mai, Siranoush Manoukian, Lesley McGuffog, Noura Mebirouk, Austin Miller, Marco Montagna, Katherine L. Nathanson, Susan L. Neuhausen, Joanne Ngeow Yuen Yie, Henriette Roed Nielsen, Liene Nikitina-Zake, Kenneth Offit, Edith Olah, Olufunmilayo I. Olopade, Laura Papi, Michael T. Parsons, Harsha Pathak, Inge Sokilde Pedersen, Ana Peixoto, Pedro Perez-Segura, Beth Peshkin, Paolo Peterlongo, Paolo Radice, Johanna Rantala, Eric Ross, Marta Santamariña, Penny Soucy, Rita K. Schmutzler, Jacques Simard, Christian F. Singer, Anna P. Sokolenko, Dominique Stoppa-Lyonnet, Yen Yen Tan, Manuel R. Teixeira, Mary Beth Terry, Mads Thomassen, Darcy L. Thull, Marc Tischkowitz, Amanda E. Toland, Diana Torres, Nadine Tung, Annemieke H. van der Hout, Elizabeth J. van Rensburg, Ana Vega, Barbara Wappenschmidt, Jeffrey N. Weitzel, Katia M. Zavaglia, Kristin K. Zorn, Thomas A. Sellers, Georgia Chenevix-Trench, Antonis C. Antoniou, Mads Thomassen, Pamela J. Thompson, Liv Cecilie Vestrheim Thomsen, Darcy L. Thull, Marc Tischkowitz, Linda Titus, Amanda E. Toland, Diana Torres, Britton Trabert, Ruth Travis, Nadine Tung, Shelley S. Tworoger, Ellen Valen, Anne M. van Altena, Annemieke H. van der Hout, Els Van Nieuwenhuysen, Elizabeth J. van Rensburg, Ana Vega, Digna Velez Edwards, Robert A. Vierkant, Frances Wang, Barbara Wappenschmidt, Penelope M. Webb, Clarice R. Weinberg, Jeffrey N. Weitzel, Nicolas Wentzensen, Emily White, Alice S. Whittemore, Stacey J. Winham, Alicja Wolk, Yin-Ling Woo, Anna H. Wu, Li Yan, Drakoulis Yannoukakos, Katia M. Zavaglia, Wei Zheng, Argyrios Ziogas, Kristin K. Zorn, Zdenek Kleibl, Douglas Easton, Kate Lawrenson, Anna DeFazio, Thomas A. Sellers, Susan J. Ramus, Celeste L. Pearce, Alvaro N. Monteiro, Julie Cunningham, Ellen L. Goode, Joellen M. Schildkraut, Andrew Berchuck, Georgia Chenevix-Trench, Simon A. Gayther, Antonis C. Antoniou, Paul D. P. Pharoah

**Affiliations:** 1 University of Cambridge, Centre for Cancer Genetic Epidemiology, Department of Public Health and Primary Care, Cambridge, UK; 2 University of Cambridge, Centre for Cancer Genetic Epidemiology, Department of Oncology, Cambridge, UK; 3 Center for Bioinformatics and Functional Genomics, Cedars-Sinai Medical Center, Los Angeles, CA USA; 4 Radboud University Medical Center, Radboud Institute for Health Sciences, Nijmegen, The Netherlands; 5 Netherlands Comprehensive Cancer Organisation, Utrecht, The Netherlands; 6 The Netherlands Cancer Institute—Antoni van Leeuwenhoek hospital, Family Cancer Clinic, Amsterdam, The Netherlands; 7 Veneto Institute of Oncology IOV—IRCCS, Immunology and Molecular Oncology Unit, Padua, Italy; 8 Lunenfeld-Tanenbaum Research Institute of Mount Sinai Hospital, Fred A. Litwin Center for Cancer Genetics, Toronto, ON Canada; 9 University of Toronto, Department of Molecular Genetics, Toronto, ON Canada; 10 University of California Irvine, Department of Epidemiology, Genetic Epidemiology Research Institute, Irvine, CA USA; 11 N.N. Alexandrov Research Institute of Oncology and Medical Radiology, Minsk, Belarus; 12 ‘Agii Anargiri’ Cancer Hospital, Athens, Greece; 13 University of Texas MD Anderson Cancer Center, Department of Breast Medical Oncology, Houston, TX USA; 14 Lund University, Department of Cancer Epidemiology, Clinical Sciences, Lund, Sweden; 15 Vall d’Hebron Institute of Oncology, Hereditary cancer Genetics Group, Barcelona, Spain; 16 University Hospital of Vall d’Hebron, Department of Medical Oncology, Barcelona, Spain; 17 Rutgers Cancer Institute of New Jersey, Cancer Prevention and Control Program, New Brunswick, NJ USA; 18 Landspitali University Hospital, Department of Pathology, Reykjavik, Iceland; 19 University of Iceland, BMC (Biomedical Centre), Faculty of Medicine, Reykjavik, Iceland; 20 University Hospital Erlangen, Friedrich-Alexander-University Erlangen-Nuremberg, Department of Gynecology and Obstetrics, Comprehensive Cancer Center ER-EMN, Erlangen, Germany; 21 Vanderbilt University School of Medicine, Division of Epidemiology, Department of Medicine, Vanderbilt Epidemiology Center, Vanderbilt-Ingram Cancer Center, Nashville, TN USA; 22 Biomedical Network on Rare Diseases (CIBERER), Madrid, Spain; 23 Spanish National Cancer Research Centre (CNIO), Human Cancer Genetics Programme, Madrid, Spain; 24 Ufa Federal Research Centre of the Russian Academy of Sciences, Institute of Biochemistry and Genetics, Ufa, Russia; 25 Princess Margaret Hospital, Division of Gynecologic Oncology, University Health Network, Toronto, ON Canada; 26 Haukeland University Hospital, Department of Obstetrics and Gynecology, Bergen, Norway; 27 University of Bergen, Centre for Cancer Biomarkers CCBIO, Department of Clinical Science, Bergen, Norway; 28 National Cancer Institute, Division of Cancer Epidemiology and Genetics, Bethesda, MD USA; 29 Hannover Medical School, Department of Radiation Oncology, Hannover, Germany; 30 Hannover Medical School, Gynaecology Research Unit, Hannover, Germany; 31 IEO, European Institute of Oncology IRCCS, Division of Cancer Prevention and Genetics, Milan, Italy; 32 Lund University and Skåne University Hospital, Department of Oncology, Lund, Sweden; 33 Cancer Research UK Cambridge Institute, University of Cambridge, Cambridge, UK; 34 Maria Sklodowska-Curie National Research Institute of Oncology, Department of Pathology and Laboratory Diagnostics, Warsaw, Poland; 35 University of Helsinki, Department of Pathology, Helsinki University Hospital, Helsinki, Finland; 36 Huntsman Cancer Institute, Department of Medicine, Salt Lake City, UT USA; 37 University Hospital, SOD Genetica Molecolare, Pisa, Italy; 38 Peter MacCallum Cancer Center, Melbourne, VIC Australia; 39 The University of Melbourne, Sir Peter MacCallum Department of Oncology, Melbourne, VIC Australia; 40 Roswell Park Cancer Institute, Cancer Pathology & Prevention, Division of Cancer Prevention and Population Sciences, Buffalo, NY USA; 41 Division of Human Genetics, The Ohio State University, Department of Internal Medicine, Columbus, OH USA; 42 German Cancer Research Center (DKFZ), Division of Cancer Epidemiology, Heidelberg, Germany; 43 University Medical Center Hamburg-Eppendorf, Cancer Epidemiology Group, University Cancer Center Hamburg (UCCH), Hamburg, Germany; 44 National Cancer Institute, National Institutes of Health, Department of Health and Human Services, Division of Cancer Epidemiology and Genetics, Bethesda, MD USA; 45 Tianjin Medical University Cancer Institute and Hospital, Department of Epidemiology, Tianjin, China; 46 The University of Sydney, Centre for Cancer Research, The Westmead Institute for Medical Research, Sydney, NSW Australia; 47 Westmead Hospital, Department of Gynaecological Oncology, Sydney, NSW Australia; 48 Columbia University, Departments of Pediatrics and Medicine, New York, NY USA; 49 Ghent University, Centre for Medical Genetics, Gent, Belgium; 50 INSERM U830, Department of Tumour Biology, Paris, France; 51 Institut Curie, Paris, France; 52 Mines ParisTech, Fontainebleau, France; 53 Faculty of Medicine and University Hospital Cologne, University of Cologne, Center for Familial Breast and Ovarian Cancer, Cologne, Germany; 54 University of New Mexico, University of New Mexico Health Sciences Center, Albuquerque, NM USA; 55 Alberta Health Services, Department of Cancer Epidemiology and Prevention Research, Calgary, AB Canada; 56 Mayo Clinic, Department of Laboratory Medicine and Pathology, Rochester, MN USA; 57 Fox Chase Cancer Center, Department of Clinical Genetics, Philadelphia, PA USA; 58 Memorial Sloan Kettering Cancer Center, Gynecology Service, Department of Surgery, New York, NY USA; 59 Cambridge, Cambridge, UK; 60 CIBERONC, Hospital Clinico San Carlos, IdISSC (Instituto de Investigación Sanitaria del Hospital Clínico San Carlos), Molecular Oncology Laboratory, Madrid, Spain; 61 NorthShore University Health System, Center for Medical Genetics, Evanston, IL USA; 62 The University of Chicago Pritzker School of Medicine, Chicago, IL USA; 63 Leiden University Medical Center, Department of Pathology, Leiden, The Netherlands; 64 Leiden University Medical Center, Department of Human Genetics, Leiden, The Netherlands; 65 Vall dHebron Institute of Oncology (VHIO), Oncogenetics Group, Barcelona, Spain; 66 University Hospital Vall dHebron, Clinical and Molecular Genetics Area, Barcelona, Spain; 67 Beckman Research Institute of City of Hope, Department of Population Sciences, Duarte, CA USA; 68 University of Utah, Huntsman Cancer Institute, Department of Population Health Sciences, Salt Lake City, UT USA; 69 University of Pennsylvania, Basser Center for BRCA, Abramson Cancer Center, Philadelphia, PA USA; 70 Ev. Kliniken Essen-Mitte (KEM), Department of Gynecology and Gynecologic Oncology, Essen, Germany; 71 Dr. Horst Schmidt Kliniken Wiesbaden, Department of Gynecology and Gynecologic Oncology, Wiesbaden, Germany; 72 Jena University Hospital—Friedrich Schiller University, Department of Gynaecology, Jena, Germany; 73 University of Southampton, Faculty of Medicine, Southampton, UK; 74 Harvard T.H. Chan School of Public Health, Department of Epidemiology, Boston, MA USA; 75 Brigham and Women’s Hospital and Harvard Medical School, Channing Division of Network Medicine, Boston, MA USA; 76 University of Leipzig, Institute for Medical Informatics, Statistics and Epidemiology, Leipzig, Germany; 77 University of Leipzig, LIFE—Leipzig Research Centre for Civilization Diseases, Leipzig, Germany; 78 University of Manchester, Manchester Academic Health Science Centre, Division of Evolution and Genomic Sciences, School of Biological Sciences, Faculty of Biology, Medicine and Health, Manchester, UK; 79 St Mary’s Hospital, Manchester University NHS Foundation Trust, Manchester Academic Health Science Centre, North West Genomics Laboratory Hub, Manchester Centre for Genomic Medicine, Manchester, UK; 80 University of California at Los Angeles, David Geffen School of Medicine, Department of Medicine Division of Hematology and Oncology, Los Angeles, CA USA; 81 Imperial College London, Division of Cancer and Ovarian Cancer Action Research Centre, Department of Surgery and Cancer, London, UK; 82 Masaryk Memorial Cancer Institute, Department of Cancer Epidemiology and Genetics, Brno, Czech Republic; 83 Chaim Sheba Medical Center, The Susanne Levy Gertner Oncogenetics Unit, Ramat Gan, Israel; 84 Tel Aviv University, Sackler Faculty of Medicine, Ramat Aviv, Israel; 85 Jonsson Comprehensive Cancer Centre, UCLA, Schools of Medicine and Public Health, Division of Cancer Prevention & Control Research, Los Angeles, CA USA; 86 Dana-Farber Cancer Institute, Cancer Risk and Prevention Clinic, Boston, MA USA; 87 University of Florence, Department of Experimental and Clinical Biomedical Sciences ‘Mario Serio’, Medical Genetics Unit, Florence, Italy; 88 Cancer Council Victoria, Cancer Epidemiology Division, Melbourne, VIC Australia; 89 The University of Melbourne, Centre for Epidemiology and Biostatistics, Melbourne School of Population and Global Health, Melbourne, VIC Australia; 90 Monash University, Precision Medicine, School of Clinical Sciences at Monash Health, Clayton, VIC Australia; 91 University of Kansas Medical Center, Department of Pathology and Laboratory Medicine, Kansas City, KS USA; 92 Cedars-Sinai Medical Center, Samuel Oschin Comprehensive Cancer Institute, Cancer Prevention and Genetics Program, Los Angeles, CA USA; 93 National Cancer Institute, Clinical Genetics Branch, Division of Cancer Epidemiology and Genetics, Bethesda, MD USA; 94 Pomeranian Medical University, Department of Genetics and Pathology, Szczecin, Poland; 95 QIMR Berghofer Medical Research Institute, Population Health Department, Brisbane, QLD Australia; 96 Faculty of Medicine and University Hospital Cologne, University of Cologne, Center for Integrated Oncology (CIO), Cologne, Germany; 97 University of Southern California, Department of Preventive Medicine, Keck School of Medicine, Los Angeles, CA USA; 98 Karolinska Institutet, Institute of Environmental Medicine, Stockholm, Sweden; 99 German Cancer Research Center (DKFZ), Molecular Genetics of Breast Cancer, Heidelberg, Germany; 100 Rigshospitalet, Copenhagen University Hospital, Department of Clinical Genetics, Copenhagen, Denmark; 101 Fred Hutchinson Cancer Research Center, Program in Epidemiology, Division of Public Health Sciences, Seattle, WA USA; 102 University of Washington, Department of Epidemiology, Seattle, WA USA; 103 National University of Singapore and National University Health System, Saw Swee Hock School of Public Health, Singapore, Singapore; 104 National University Health System, Department of Surgery, Singapore, Singapore; 105 Humboldt-Universität zu Berlin, and Berlin Institute of Health, Department for Gynecology with the Center for Oncologic Surgery Charité Campus Virchow-Klinikum, Charité – Universitätsmedizin Berlin, corporate member of Freie Universität Berlin, Berlin, Germany; 106 University of Texas MD Anderson Cancer Center, Department of Epidemiology, Houston, TX USA; 107 Danish Cancer Society Research Center, Department of Virus, Lifestyle and Genes, Copenhagen, Denmark; 108 University of Copenhagen, Molecular Unit, Department of Pathology, Herlev Hospital, Copenhagen, Denmark; 109 University of Copenhagen, Department of Gynaecology, Rigshospitalet, Copenhagen Denmark; 110 Roswell Park Cancer Institute, Center For Immunotherapy, Buffalo, NY USA; 111 BC Cancer, Vancouver General Hospital, and University of British Columbia, British Columbia’s Ovarian Cancer Research (OVCARE) Program, Vancouver, BC Canada; 112 University of British Columbia, Department of Pathology and Laboratory Medicine, Vancouver, BC Canada; 113 University of British Columbia, Department of Obstetrics and Gynecology, Vancouver, BC Canada; 114 BC Cancer Research Centre, Department of Molecular Oncology, Vancouver, BC Canada; 115 N.N. Petrov Institute of Oncology, St. Petersburg, Russia; 116 Coordinating center: The Netherlands Cancer Institute, The Hereditary Breast and Ovarian Cancer Research Group Netherlands (HEBON), Amsterdam, The Netherlands; 117 Lombardi Comprehensive Cancer Center, Georgetown University, Washington, DC USA; 118 Pomeranian Medical University, Independent Laboratory of Molecular Biology and Genetic Diagnostics, Szczecin, Poland; 119 Peter MacCallum Cancer Center, Parkville Familial Cancer Centre, Melbourne, VIC Australia; 120 Vilnius University Hospital Santariskiu Clinics, Hematology, oncology and transfusion medicine center, Dept. of Molecular and Regenerative Medicine, Vilnius, Lithuania; 121 State Research Institute Centre for Innovative Medicine, Vilnius, Lithuania; 122 Landspitali University Hospital, Department of Oncology, Reykjavik, Iceland; 123 Stanford University School of Medicine, Department of Epidemiology & Population Health, Stanford, CA USA; 124 Stanford Cancer Institute, Stanford University School of Medicine, Department of Medicine, Division of Oncology, Stanford, CA USA; 125 The Institute of Cancer Research, Division of Genetics and Epidemiology, London, UK; 126 Seoul National University College of Medicine, Department of Preventive Medicine, Seoul, Korea; 127 Seoul National University Graduate School, Department of Biomedical Sciences, Seoul, Korea; 128 Seoul National University, Cancer Research Institute, Seoul, Korea; 129 University of California at Los Angeles, David Geffen School of Medicine, Department of Obstetrics and Gynecology, Los Angeles, CA USA; 130 UC Davis Medical Center, Department of Pathology and Laboratory Medicine, Sacramento, CA USA; 131 Medical University of South Carolina, Hollings Cancer Center, Charleston, SC USA; 132 Saint Petersburg State University, Saint Petersburg, Russia; 133 Sungkyunkwan University School of Medicine, Department of Obstetrics and Gynecology, Samsung Medical Center, Seoul, Korea; 134 City of Hope Clinical Cancer Genetics Community Research Network, Duarte, CA USA; 135 Cancer Genetics Centre, Hong Kong Hereditary Breast Cancer Family Registry, Happy Valley, Hong Kong; 136 The University of Hong Kong, Department of Surgery, Pok Fu Lam, Hong Kong; 137 Hong Kong Sanatorium and Hospital, Department of Surgery, Happy Valley, Hong Kong; 138 VIB Center for Cancer Biology, Leuven, Belgium; 139 University of Leuven, Laboratory for Translational Genetics, Department of Human Genetics, Leuven, Belgium; 140 Mayo Clinic, Department of Health Sciences Research, Division of Biomedical Statistics and Informatics, Rochester, MN USA; 141 ONCOBELL-IDIBELL-IGTP, Catalan Institute of Oncology, CIBERONC, Hereditary Cancer Program, Barcelona, Spain; 142 BC Cancer, Cancer Control Research, Vancouver, BC Canada; 143 Inserm U900, Genetic Epidemiology of Cancer team, Paris, France; 144 NYU Langone Medical Center, Gynecologic Oncology, Laura and Isaac Pearlmutter Cancer Center, New York, NY USA; 145 Genome Institute of Singapore, Human Genetics Division, Singapore, Singapore; 146 University of Texas MD Anderson Cancer Center, Department of Gynecologic Oncology and Clinical Cancer Genetics Program, Houston, TX USA; 147 Magee-Womens Hospital, University of Pittsburgh School of Medicine, Pittsburgh, PA USA; 148 Fondazione IRCCS Istituto Nazionale dei Tumori di Milano, Unit of Medical Genetics, Department of Medical Oncology and Hematology, Milan, Italy; 149 Duke University Hospital, Department of Surgery, Durham, NC USA; 150 University of Hawaii Cancer Center, Cancer Epidemiology Program, Honolulu, HI USA; 151 Aichi Cancer Center Research Institute, Division of Cancer Epidemiology and Prevention, Nagoya, Japan; 152 Nagoya University Graduate School of Medicine, Division of Cancer Epidemiology, Nagoya, Japan; 153 Samuel Lunenfeld Research Institute, Public Health Ontario, Toronto, ON Canada; 154 Imperial College London, Division of Cancer and Ovarian Cancer Action Research Centre, Department Surgery & Cancer, London, UK; 155 University of Glasgow, Institute of Cancer Sciences, Glasgow, UK; 156 University College London, MRC Clinical Trials Unit at UCL, Institute of Clinical Trials & Methodology, London, UK; 157 Roswell Park Cancer Institute, NRG Oncology, Statistics and Data Management Center, Buffalo, NY USA; 158 Roswell Park Cancer Institute, Division of Cancer Prevention and Control, Buffalo, NY USA; 159 Magee-Womens Research Institute and Hillman Cancer Center, Womens Cancer Research Center, Pittsburgh, PA USA; 160 University of Pittsburgh School of Medicine, Division of Gynecologic Oncology, Department of Obstetrics, Gynecology and Reproductive Sciences, Pittsburgh, PA USA; 161 Oregon Health & Science University, Department of Obstetrics and Gynecology, Portland, OR USA; 162 Oregon Health & Science University, Knight Cancer Institute, Portland, OR USA; 163 University of Helsinki, Department of Obstetrics and Gynecology, Helsinki University Hospital, Helsinki, Finland; 164 National Cancer Centre, Cancer Genetics Service, Singapore, Singapore; 165 Nanyang Technological University, Lee Kong Chian School of Medicine, Singapore, Singapore; 166 Odense University Hospital, Department of Clinical Genetics, Odence C, Denmark; 167 Latvian Biomedical Research and Study Centre, Riga, Latvia; 168 Roswell Park Cancer Institute, Department of Gynecologic Oncology, Buffalo, NY USA; 169 Memorial Sloan Kettering Cancer Center, Clinical Genetics Research Lab, Department of Cancer Biology and Genetics, New York, NY USA; 170 Memorial Sloan Kettering Cancer Center, Clinical Genetics Service, Department of Medicine, New York, NY USA; 171 National Institute of Oncology, Department of Molecular Genetics, Budapest, Hungary; 172 University Hospitals Leuven, Division of Gynecologic Oncology, Department of Obstetrics and Gynaecology and Leuven Cancer Institute, Leuven, Belgium; 173 The University of Chicago, Center for Clinical Cancer Genetics, Chicago, IL USA; 174 Memorial Sloan-Kettering Cancer Center, Department of Epidemiology and Biostatistics, New York, NY USA; 175 Centro de Investigación en Red de Enfermedades Raras (CIBERER), Madrid, Spain; 176 QIMR Berghofer Medical Research Institute, Department of Genetics and Computational Biology, Brisbane, QLD Australia; 177 Aalborg University Hospital, Molecular Diagnostics, Aalborg, Denmark; 178 Aalborg University Hospital, Clinical Cancer Research Center, Aalborg, Denmark; 179 Aalborg University, Department of Clinical Medicine, Aalborg, Denmark; 180 Portuguese Oncology Institute, Department of Genetics, Porto, Portugal; 181 Moffitt Cancer Center, Department of Cancer Epidemiology, Tampa, FL USA; 182 IFOM—the FIRC Institute of Molecular Oncology, Genome Diagnostics Program, Milan, Italy; 183 Bashkir State University, Department of Genetics and Fundamental Medicine, Ufa, Russia; 184 Fondazione IRCCS Istituto Nazionale dei Tumori (INT), Unit of Molecular Bases of Genetic Risk and Genetic Testing, Department of Research, Milan, Italy; 185 Karolinska Institutet, Clinical Genetics, Stockholm, Sweden; 186 Duke University Hospital, Department of Gynecologic Oncology, Durham, NC USA; 187 Yale School of Public Health, Chronic Disease Epidemiology, New Haven, CT USA; 188 Fox Chase Cancer Center, Population Studies Facility, Philadelphia, PA USA; 189 National Institute of Environmental Health Sciences, NIH, Epidemiology Branch, Research Triangle Park, NC USA; 190 Fundación Pública Galega Medicina Xenómica, Santiago De Compostela, Spain; 191 Instituto de Investigación Sanitaria de Santiago de Compostela, Santiago De Compostela, Spain; 192 Centre Hospitalier Universitaire de Québec – Université Laval Research Center, Genomics Center, Québec City, QC Canada; 193 Faculty of Medicine and University Hospital Cologne, University of Cologne, Center for Molecular Medicine Cologne (CMMC), Cologne, Germany; 194 Hebei Medical University, Fourth Hospital, Department of Obstetrics and Gynaecology, Shijiazhuang, China; 195 Icahn School of Medicine at Mount Sinai, Department of Population Health Science and Policy, New York, NY USA; 196 Icahn School of Medicine at Mount Sinai, Department of Genetics and Genomic Sciences, New York, NY USA; 197 Centre Hospitalier Universitaire de Québec-Université Laval Research Center, Genomic Center, Québec City, QC Canada; 198 Medical University of Vienna, Dept of OB/GYN and Comprehensive Cancer Center, Vienna, Austria; 199 University of Cambridge, Department of Public Health and Primary Care, Cambridge, UK; 200 The University of Melbourne, Department of Clinical Pathology, Melbourne, VIC Australia; 201 Royal Alexandra Hospital, Department of Obstetrics and Gynecology, Division of Gynecologic Oncology, Edmonton, AB Canada; 202 Institut Curie, Service de Génétique, Paris, France; 203 Université Paris Descartes, Paris, France; 204 University of South Florida, Epidemiology Center, College of Medicine, Tampa, FL USA; 205 The Institute of Cancer Research, Division of Breast Cancer Research, London, UK; 206 University of Porto, Biomedical Sciences Institute (ICBAS), Porto, Portugal; 207 Cancer Research Malaysia, Breast Cancer Research Programme, Subang Jaya, Selangor Malaysia; 208 University of Malaya, Department of Surgery, Faculty of Medicine, Kuala Lumpur, Malaysia; 209 Brigham and Women’s Hospital and Harvard Medical School, Obstetrics and Gynecology Epidemiology Center, Boston, MA USA; 210 Columbia University, Department of Epidemiology, Mailman School of Public Health, New York, NY USA; 211 Magee-Womens Hospital, University of Pittsburgh School of Medicine, Department of Medicine, Pittsburgh, PA USA; 212 McGill University, Program in Cancer Genetics, Departments of Human Genetics and Oncology, Montréal, QC Canada; 213 University of Cambridge, Department of Medical Genetics, Cambridge, UK; 214 Dartmouth College, Geisel School of Medicine, Hanover, NH USA; 215 The Ohio State University, Department of Cancer Biology and Genetics, Columbus, OH USA; 216 Pontificia Universidad Javeriana, Institute of Human Genetics, Bogota, Colombia; 217 University of Oxford, Cancer Epidemiology Unit, Oxford, UK; 218 Beth Israel Deaconess Medical Center, Department of Medical Oncology, Boston, MA USA; 219 University Medical Center Groningen, University Groningen, Department of Genetics, Groningen, The Netherlands; 220 University of Pretoria, Department of Genetics, Arcadia, South Africa; 221 Fundación Pública Galega de Medicina Xenómica, Santiago de Compostela, Spain; 222 Instituto de Investigación Sanitaria de Santiago de Compostela (IDIS), Complejo Hospitalario Universitario de Santiago, SERGAS, Santiago de Compostela, Spain; 223 Vanderbilt University Medical Center, Division of Quantitative Sciences, Department of Obstetrics and Gynecology, Department of Biomedical Sciences, Women’s Health Research, Nashville, TN USA; 224 Duke Cancer Institute, Cancer Control and Population Sciences, Durham, NC USA; 225 Duke University Hospital, Department of Community and Family Medicine, Durham, NC USA; 226 National Institute of Environmental Health Sciences, NIH, Biostatistics and Computational Biology Branch, Research Triangle Park, NC USA; 227 City of Hope, Clinical Cancer Genomics, Duarte, CA USA; 228 Fred Hutchinson Cancer Research Center, Seattle, WA USA; 229 Stanford University School of Medicine, Department of Biomedical Data Science, Stanford, CA USA; 230 Uppsala University, Department of Surgical Sciences, Uppsala, Sweden; 231 University of Malaya, Department of Obstetrics and Gynaecology, University of Malaya Medical Centre, Kuala Lumpur, Malaysia; 232 Hebei Medical University, Fourth Hospital, Department of Molecular Biology, Shijiazhuang, China; 233 National Centre for Scientific Research ‘Demokritos’, Molecular Diagnostics Laboratory, INRASTES, Athens, Greece; 234 Institute of Biochemistry and Experimental Oncology, First Faculty od Medicine, Charles University, Prague, Czech Republic; 235 Women’s Cancer Program at the Samuel Oschin Comprehensive Cancer Institute, Cedars-Sinai Medical Centre, Department of Obstetrics and Gynecology, Los Angeles, CA USA; 236 Royal Pass Road, Tampa, FL USA; 237 University of NSW Sydney, School of Women’s and Children’s Health, Faculty of Medicine, Sydney, NSW Australia; 238 University of NSW Sydney, Adult Cancer Program, Lowy Cancer Research Centre, Sydney, NSW Australia; 239 University of Michigan School of Public Health, Department of Epidemiology, Ann Arbor, MI USA; 240 University of Southern California Norris Comprehensive Cancer Center, Department of Preventive Medicine, Keck School of Medicine, Los Angeles, CA USA; 241 Mayo Clinic, Department of Health Science Research, Division of Epidemiology, Rochester, MN USA; 242 Emory University, Department of Epidemiology, Rollins School of Public Health, Atlanta, GA USA

**Keywords:** Risk factors, Clinical genetics, Genetic markers

## Abstract

Polygenic risk scores (PRS) for epithelial ovarian cancer (EOC) have the potential to improve risk stratification. Joint estimation of Single Nucleotide Polymorphism (SNP) effects in models could improve predictive performance over standard approaches of PRS construction. Here, we implemented computationally efficient, penalized, logistic regression models (lasso, elastic net, stepwise) to individual level genotype data and a Bayesian framework with continuous shrinkage, “select and shrink for summary statistics” (S4), to summary level data for epithelial non-mucinous ovarian cancer risk prediction. We developed the models in a dataset consisting of 23,564 non-mucinous EOC cases and 40,138 controls participating in the Ovarian Cancer Association Consortium (OCAC) and validated the best models in three populations of different ancestries: prospective data from 198,101 women of European ancestries; 7,669 women of East Asian ancestries; 1,072 women of African ancestries, and in 18,915 *BRCA1* and 12,337 *BRCA2* pathogenic variant carriers of European ancestries. In the external validation data, the model with the strongest association for non-mucinous EOC risk derived from the OCAC model development data was the S4 model (27,240 SNPs) with odds ratios (OR) of 1.38 (95% CI: 1.28–1.48, AUC: 0.588) per unit standard deviation, in women of European ancestries; 1.14 (95% CI: 1.08–1.19, AUC: 0.538) in women of East Asian ancestries; 1.38 (95% CI: 1.21–1.58, AUC: 0.593) in women of African ancestries; hazard ratios of 1.36 (95% CI: 1.29–1.43, AUC: 0.592) in *BRCA1* pathogenic variant carriers and 1.49 (95% CI: 1.35–1.64, AUC: 0.624) in *BRCA2* pathogenic variant carriers. Incorporation of the S4 PRS in risk prediction models for ovarian cancer may have clinical utility in ovarian cancer prevention programs.

## Introduction

Rare variants in known high and moderate penetrance susceptibility genes (*BRCA1*, *BRCA2*, *BRIP1*, *PALB2*, *RAD51C*, *RAD51D* and the mis-match repair genes) account for about 40% of the inherited component of EOC disease risk [1, 2]. Common susceptibility variants, reviewed in Kar et al. and Jones et al., explain about 6% of the heritability of EOC [1, 3]. Polygenic risk scores (PRS) provide an opportunity for refined risk stratification in the general population and in carriers of rare moderate or high risk alleles.

A PRS is calculated as the weighted sum of the number of risk alleles carried for a specified set of variants. The best approach to identify the variant set and their weights to optimize the predictive power of a PRS is unknown. A common approach involves selecting a set of variants that reach a threshold for association based on the *p*-value for each variant with or without pruning to remove highly correlated variants [4, 5]. More complex machine learning approaches that do not assume variant independence have also been used [6, 7], but these methods have produced only modest gains in predictive power for highly polygenic phenotypes [6, 8]. Penalized regression approaches such as the lasso, elastic net and the adaptive lasso have also been used with individual level data [9], but a major drawback is the computational burden required to fit the models [9, 10].

We present novel, computationally efficient PRS models using two approaches: (1) penalized regression models including the lasso, elastic net and minimax concave penalty (MCP) for use with individual genotype data; and (2) a Bayesian regression model with continuous shrinkage priors for use where only summary statistics are available—referred to as the “select and shrink with summary statistics” (S4) method. We compare these models with two commonly used methods, stepwise regression with *p*-value thresholding and LDPred.

## Materials (subjects) and methods

### Model development study population

EOC is a highly heterogeneous phenotype with five major histotypes for invasive disease—high-grade serous, low-grade serous, endometrioid, clear cell, and mucinous histotype. The mucinous histotype is the least common and its origin is the most controversial with up to 60% of diagnosed cases of mucinous ovarian cancer often being misdiagnosed metastasis from non-ovarian sites [11]. Therefore, in this study, we performed PRS modeling and association testing for all cases of invasive, non-mucinous EOC. We used genotype data from 23,564 invasive non-mucinous EOC cases and 40,138 controls with >80% European ancestries from 63 case-control studies included in the Ovarian Cancer Association Consortium (OCAC) for model development. The distribution of cases by histotype was high-grade serous (13,609), low-grade serous (2,749), endometrioid (2,877), clear cell (1,427), and others (2,902). Sample collection, genotyping, and quality control have been previously described [12]. Genotype data were imputed to the Haplotype Reference Consortium reference panel using 470,825 SNPs that passed quality control. Of the 32 million SNPs imputed, 10 million had imputation *r*^2^ > 0.3 and were included in this analysis.

### Model validation study populations

We validated the best-fitting PRS models developed in the OCAC data in 657 prevalent and incident cases of invasive, non-mucinous EOC and 198,101 female controls of European ancestries from the UK Biobank. Samples were genotyped using either the Affymetrix UK BiLEVE Axiom Array or Affymetrix UK Biobank Axiom Array (which share 95% marker content), and then imputed to a combination of the Haplotype Reference Consortium, the 1000 Genomes phase 3 and the UK10K reference panels [13]. We restricted analysis to genetically confirmed females of European ancestries. We excluded individuals if they were outliers for heterozygosity, had low genotyping call rate <95%, had sex chromosome aneuploidy, or if they were duplicates (cryptic or intended) [12]. All SNPs selected in the model development phase were available in the UK Biobank.

We investigated transferability of the best-fitting PRS models to populations of non-European ancestries using genotype data from females of East Asian and African ancestries genotyped as part of the OCAC OncoArray Project [14, 15]. Women of East Asian ancestries—2,841 non-mucinous invasive EOC and 4,828 controls—were identified using a criterion of >80% Asian ancestries. This included samples collected from studies in China, Japan, Korea, and Malaysia as well as samples collected from women of Asian ancestry in studies conducted in the US, Europe and Australia [14]. Similarly, women of African ancestries—368 cases of non-mucinous invasive EOC and 704 controls—mainly from studies conducted in the US, were identified using a criterion of >80% African ancestries as described previously [15].

We also assessed the performance of the best-fitting PRS models in women of European ancestries (>80% European ancestries) with the pathogenic *BRCA1* and *BRCA2* variants from the Consortium of Investigators of Modifiers of *BRCA1/2* (CIMBA). We used genotype data from 18,915 *BRCA1* (2,053 invasive EOC cases) and 12,337 *BRCA2* (717 invasive EOC cases) pathogenic variant carriers from 63 studies contributing to CIMBA [16]. Genotyping, data quality control measures, intercontinental ancestries assessment and imputation to the HRC reference panel are as described for the OCAC study population.

## Statistical analysis

### Polygenic risk models

For all PRS models, we created scores as linear functions of the allele dosage in the general form $PRS_i = \mathop {\sum}\nolimits_j^p {x_{ij}\beta _j}$ where genotypes are denoted as *x* (taking on the minor allele dosages of 0, 1, and 2), with *x*_*ij*_ representing the *i*th individual for the *j*th SNP (out of *p* SNPs) on an additive log scale and *β*_*j*_ represents the weight—the log of the odds ratio—of the *j*th SNP. We used different approaches to select and derive the optimal weights, *β*_*j*_, in models as described below.

### Penalized logistic regression models

A penalized logistic regression model for a set of SNPs aims to identify a set of regression coefficients that minimize the regularized loss function given by

$$plr\left( {x;\lambda ,\kappa } \right) = \left\{ {\begin{array}{*{20}{c}} {x - \lambda sign\left( x \right)/\left( {1 - \kappa } \right)\,if\left| x \right| \, < \, \lambda /\kappa \,and\,\left| {\left( x \right)} \right| \, > \, \lambda } \\ {x\,if\left| x \right| \ge \frac{\lambda }{\kappa }} \\ {0\,if\left| {\left( x \right)} \right| \, < \, \lambda } \end{array}} \right.$$

where *x* is the effect estimate of a SNP, *λ* is the tuning parameter and *κ* is the threshold (penalty) for different regularization paths. *λ* and *κ* are parameters that need to be chosen during model development to optimize performance. The lasso, elastic net, MCP, and *p*-value thresholds are instances of the function with different *κ* values. We minimized the winner’s curse effect on inflated effect estimates for rare SNPs by penalizing rarer SNPs more heavily than common SNPs. Details are provided in the Supplementary Methods.

We used a two-stage approach to reduce computational burden without a corresponding loss in predictive power. The first stage was a SNP selection stage using a sliding windows approach, with 5.5 Mb data blocks and a 500 kb overlap between blocks. SNP selection was performed for each block and selected SNPs were collated. Single SNP association analyses were then run, and all SNPs with a *χ*^2^ test statistic of less than 2.25 were excluded. The 2.25 cutoff was arbitrary and selected to maximize computational efficiency without loss in predictive power. Penalized regression models were applied to the remaining SNPs using *λ* values of 3.0 and *κ* values of 0.0, 0.2, 0.4, 0.6, 0.8 and 1.0. SNPs selected in any of these models were included in subsequent analyses. In the second stage, we fit penalized regression models to the training dataset with *λ* values ranging from 3.0 to 5.5 in increments of 0.1 iterated over *κ* values from −3.0 to 1 in increments of 0.1. The lasso model (*κ* = 0) for each value of *λ* was fitted first, to obtain a unique maximum. From the fitted maximum the *κ* value was changed, and the model refitted.

**Fig. 1:**
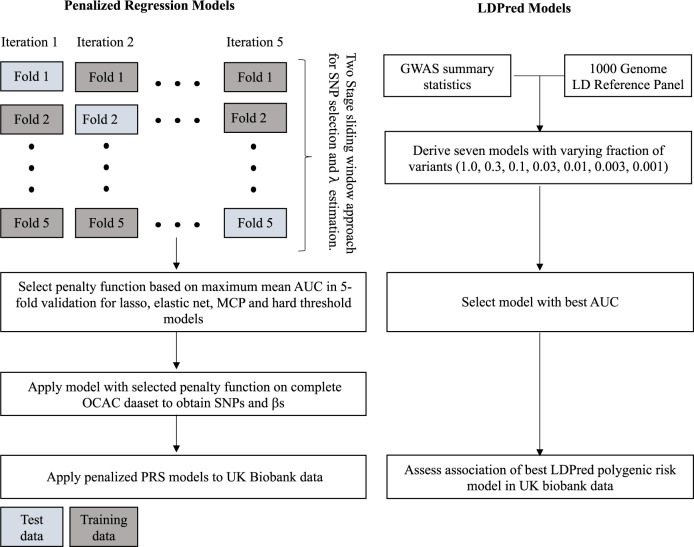
PRS model development using penalized regression and LDPred Bayesian approach. Shown in the left panel is the two-stage approach with five-fold cross validation used for individual level genotype data while the right panel shows the LDPred approach used for summary level data.

We applied this two-stage approach with five-fold cross-validation (Fig. 1). In each iteration, the data set was split into five, with one part constituting the test data and the other four constituting the training data. The variants and their weights from the two-stage penalized logistic regression modeling in the training data were used to calculate the area under the receiver operating characteristic curve (AUC) in the test data in each iteration. AUC estimates for each combination of *λ* and *κ* were obtained. We repeated this process for each cross-validation iteration to obtain a mean AUC for each combination of *λ* and *κ*. Finally, we selected the tuning and threshold parameters from the lasso, elastic net and MCP models with the maximum mean cross-validated AUC and fitted penalized logistic regression models with these parameters to the entire OCAC dataset to obtain SNP weights for PRS scores.

### Stepwise logistic regression with variable *P*-value threshold

This model is a general PLR model with *κ* = 1. As with the other PLR models, we investigated various values for *λ* values (corresponding to a variable *P*-value threshold for including a SNP in the model). However, we observed that the implementation of this model on individual level data was more difficult than for other *κ* values because the model would sometimes converge to a local optimum rather than the global optimum. Therefore, we applied an approximate conditional and joint association analysis using summary level statistics correcting for estimated LD between SNPs, and utilizing  a reference panel of 5,000 individual level genotype OCAC data as described in Yang et al. [17]. Details are provided in the Supplementary Methods.

### LDPred

LDPred is a Bayesian approach that shrinks the posterior mean effect size of each marker based on a point-normal prior and LD information from an external reference panel. We derived seven candidate PRSs assuming the fractions of associated variants were 0.001, 0.003, 0.01, 0.03, 0.1, 0.3, and 1.0 respectively using the default parameters as detailed in Vilhjálmsson et al. [18] and an LD reference panel of 503 samples of European ancestries from the 1000 Genomes phase 3 release with effect estimates from the OCAC model development data.

### Select and shrink using summary statistics (S4)

The S4 algorithm is similar to the PRS-CS algorithm [19]—a Bayesian method that uses summary statistics and between-SNP correlation data from a reference panel to generate the PRS scores by placing a continuous shrinkage prior on effect sizes. We adapted this method with penalization of rarer SNPs by correcting for the standard deviation resulting in the selection of fewer SNPs. We varied three parameters, *a, b*, φ, which control the degree of shrinkage of effect estimates. Φ, the overall shrinkage parameter, is influenced by values of *a* which controls shrinkage of effect estimates around 0 and *b* which control shrinkage of larger effect estimates. We generated summary statistics for each cross-validation training set and selected the parameters that gave the best results on average from the cross-validation and applied these to the set of summary statistics for the complete OCAC data set to obtain the final set of weights.

### PRS based on meta-analysis of OCAC-CIMBA summary statistics

We conducted a meta-analysis of the EOC associations in *BRCA1* variant carriers, *BRCA2* variant carriers and the participants participating in OCAC (see Supplementary Methods) and constructed two PRS models. An S4 PRS was generated by applying the *a, b* and φ parameters from the S4 model described above. A stepwise PRS was generated by selecting all SNPs that were genome-wide significant (*p* < 5 × 10^−8^) in the meta-analysis, along with any independent signals in the same region with *p* < 10^−5^ from the histotype specific analyses for low-grade serous, high-grade serous, endometrioid, clear cell ovarian cancer and non-mucinous invasive EOC.

### Polygenic risk score performance

The best lasso, elastic net, stepwise and S4 models from the model development stage were validated using two independent data sources: the UK Biobank data and *BRCA1/BRCA2* pathogenic variant carriers from the CIMBA. In the UK Biobank data, we evaluated discriminatory performance of the models using the AUC and examined the association between standardized PRS and risk of non-mucinous EOC using logistic regression analysis. For the CIMBA data, we assessed associations for each version of the PRS and invasive non-mucinous EOC risk using weighted Cox regression methods [20]. PRSs in the CIMBA data were scaled to the same PRS standard deviations as the OCAC data, meaning that per standard deviation hazard ratios estimated on CIMBA data are comparable to PRS associations in the OCAC and UK Biobank data. The regression models were adjusted for birth cohort (<1920, 1920–1929, 1930–1939, 1940–1949, ≥1950) and the first four ancestries informative principal components (calculated separately by iCOGS/OncoArray genotyping array) and stratified by Ashkenazi Jewish ancestries and country. Absolute risks by PRS percentiles adjusting for competing risks of mortality from other causes were calculated as described in the Supplementary Material.

### Transferability of PRS scores to non-European ancestries

We implemented two straightforward approaches to disentangle the role of ancestries on polygenic risk scoring. We selected homogenous ancestral samples by using a high cut-off criterion of 80% ancestries and we standardized the PRSs by mean-centering within each population. These approaches led to a more uniform distribution of PRSs within each ancestral population. Further adjustments using principal components of ancestries did not attenuate risk estimates.

## Results

### Model development

The results for the models based on individual level genotype data are shown in Table 1. The elastic net model had the best predictive accuracy (AUC = 0.586). The optimal value of λ obtained from regularization paths for the MCP model was 3.3 meaning the best MCP model was equivalent to the lasso model. The best-fitting model based on summary statistics was the S4 (AUC = 0.593) and the LDPred model had the poorest performance of the methods tested (AUC = 0.552). Therefore, the LDPred model was not considered for further validation in other datasets. All SNPs selected and the associated weights for each model are provided in Supplementary Tables 1–6.

**Table 1:** Performance of different PRS models in five-fold cross-validation of OCAC data.

Model	Number of SNPs^a^	Tuning parameter for best performance	AUC	OR per 1 SD of PRS	95% CI
(a) Models based on individual level genotype data					
Lasso	1403	λ = 3.3	0.583	1.35	1.30–1.39
Elastic net	10,797	λ = 3.3, *κ* = −2.2	0.586	1.36	1.31–1.40
MCP	1403	λ = 3.3	0.583	1.35	1.30–1.39
(b) Models based on summary statistics					
LDPred	5,291,719	*ρ* = 0.001	0.552	1.21	1.13–1.29
Stepwise	22	λ = 5.4	0.572	1.30	1.26–1.34
Select and Shrink (OCAC)	27,240	a = 2.75, b = 2, φ = 3e−6	0.593	1.39	1.34–1.44

*AUC* area under the receiver operating characteristic (ROC) curve AUC), *OR* odds ratio, *SD* standard deviation, *PRS* polygenic risk score, *CI* confidence interval, *NA* not applicable.

^a^Number of SNPs in PRS model run on full OCAC data set after selection of model parameters.

### Model validation in women of European ancestries

Overall the PLR models performed slightly better in the UK Biobank data than the model development data (Table 2). Of the models developed using the OCAC model development data, the association was strongest with the S4 PRS. In *BRCA1* and *BRCA2* variant carriers, prediction accuracy was generally higher among *BRCA2* carriers than *BRCA1* carriers. Consistent with results from the general population in the UK Biobank, the S4 PRS model also had the strongest association and predictive accuracy for invasive EOC risk in both *BRCA1* and *BRCA2* carriers. Sensitivity analyses were conducted in which the unadjusted models for *BRCA1* and *BRCA2* carriers were progressively adjusted for birth cohort and 6 principal components. There was little difference in HR estimates and association *P*-values going from the unadjusted model to the model adjusting for six principal components (Supplementary Table 7). The PRS models developed using the OCAC-CIMBA meta-analysis results had better discriminative ability in the UK Biobank than the PRS models developed using only OCAC data. Compared with the S4 PRS using only OCAC data, the S4 PRS model derived from the meta-analysis had fewer SNPs, a stronger association with invasive EOC risk and better predictive accuracy. Similarly, the stepwise model from the OCAC-CIMBA meta-analysis performed better than the stepwise model from only OCAC data, but included more SNPs.

**Table 2:** External validation of PRS models in European populations using data from UK Biobank and CIMBA.

Model (data set)	SNPs	UK Biobank			CIMBA *BRCA1* carriers^a^			CIMBA *BRCA2* carriers^a^			
		AUC	OR	95% CI	AUC	HR	95% CI	AUC	HR	95% CI	
(a) PRS models based on OCAC data											
Lasso (OCAC)	1403	0.587	1.37	1.27–1.48	0.573	1.27	1.21–1.34	0.627	1.48	1.33–1.63	
Elastic net (OCAC)	10,797	0.588	1.36	1.26–1.47	0.583	1.32	1.26–1.39	0.617	1.47	1.33–1.63	
Stepwise (OCAC)	22	0.588	1.35	1.26–1.46	0.563	1.21	1.16–1.26	0.605	1.39	1.26–1.54	
Select and shrink (OCAC)	27,240	0.588	1.38	1.28–1.48	0.592	1.36	1.29–1.43	0.624	1.49	1.35–1.64	
(b) PRS models based on meta-analysis of OCAC and CIMBA data											
Stepwise (OCAC-CIMBA)^b^	36	0.595	1.39	1.29–1.50	NA	NA	NA	NA	NA	NA	
Select and shrink (OCAC-CIMBA)	18,007	0.596	1.42	1.32–1.54	NA	NA	NA	NA	NA	NA	

*AUC* area under the receiver operating characteristic curve, *OR* odds ratio, *HR* hazards ratio.

^a^Estimates are from unadjusted models.

^b^Results in CIMBA are overfitted as the CIMBA data was used for model development.

**Fig. 2:**
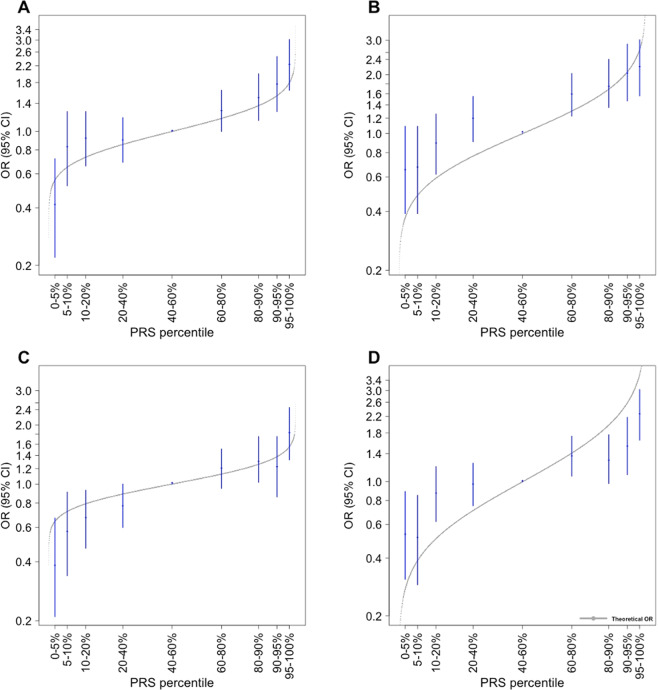
Association between the PLR PRS models and non-mucinous ovarian cancer by PRS percentiles. Shown are estimated odds ratios (OR) and confidence intervals for women of European ancestries by percentiles of polygenic risk scores derived from lasso (**A**), elastic net (**B**), stepwise (**C**) and S4 (**D**) models relative to the middle quintile.

The observed distribution of the OR estimates within centiles of the PRS distribution were consistent with ORs from predicted values under the assumption that all SNPs interact multiplicatively (Fig. 2), with all 95% confidence intervals intersecting with the theoretical estimates for women of European ancestries. Compared with women in the middle quintile, women of European ancestry (UK Biobank) in the top 95th percentile of the lasso derived PRS model had a 2.23-fold increased odds of non-mucinous EOC (95% CI: 1.64 - 3.02) (Table 3).

**Table 3:** Association between polygenic risk scores and non-mucinous EOC by PRS percentiles and ancestry.

	UK Biobank			East Asian			African			
Percentile	Controls (*n*)	Cases (*n*)	OR (95% CI)	Controls (*n*)	Cases (*n*)	OR (95% CI)	Controls (*n*)	Cases (*n*)	OR (95% CI)	
(a) Lasso										
0–5	9880	12	0.42 (0.22–0.72)	278	106	0.65 (0.51–0.83)	35	19	0.89 (0.47–1.65)	
5–10	9870	24	0.83 (0.52–1.27)	271	112	0.71 (0.55–0.90)	41	13	0.52 (0.25–1.01)	
10–20	19,733	53	0.92 (0.66–1.27)	487	280	0.98 (0.82–1.18)	81	26	0.53 (0.31–0.88)	
20–40	39,468	104	0.90 (0.69–1.18)	993	541	0.93 (0.80–1.08)	154	60	0.64 (0.42–0.99)	
40–60	39,457	115	1	967	566	1	133	81	1	
60–80	39,425	147	1.28 (1.00–1.64)	941	593	1.08 (0.93–1.25)	136	78	0.94 (0.64–1.39)	
80–90	19,699	87	1.52 (1.14–2.00)	466	301	1.10 (0.92–1.32)	63	44	1.15 (0.71–1.84)	
90–95	9842	51	1.78 (1.27–2.46)	214	169	1.35 (1.07–1.69)	34	20	0.97 (0.51–1.78)	
95–100	9830	64	2.23 (1.64–3.02)	211	173	1.40 (1.12–1.76)	27	27	1.64 (0.90–3.00)	
(b) Elastic net										
0–5	9876	17	0.67 (0.39–1.09)	277	107	0.72 (0.56–0.92)	35	19	0.90 (0.47–1.64)	
5–10	9876	17	0.67 (0.39–1.09)	271	112	0.78 (0.61–0.99)	41	13	0.52 (0.25–1.01)	
10–20	19,740	45	0.89 (0.62–1.26)	497	270	1.02 (0.85–1.22)	81	26	0.53 (0.31–0.88)	
20–40	39,453	120	1.19 (0.91–1.55)	967	567	1.10 (0.95–1.28)	154	60	0.64 (0.42–0.96)	
40–60	39,471	101	1	1000	533	1	133	81	1	
60–80	39,413	159	1.58 (1.23–2.03)	926	608	1.23 (1.06–1.43)	136	78	0.94 (0.64–1.39)	
80–90	19,695	91	1.80 (1.36–2.40)	457	310	1.27 (1.06–1.52)	63	44	1.15 (0.71–1.84)	
90–95	9841	52	2.07 (1.47–2.87)	226	157	1.30 (1.04–1.64)	34	20	0.97 (0.51–1.78)	
95–100	9839	55	2.18 (1.56–3.02)	207	177	1.60 (1.28–2.01)	27	27	1.64 (0.90–3.00)	
(c) Stepwise										
0–5	9880	13	0.39 (0.21–0.67)	254	130	0.90 (0.71–1.14)	40	14	0.75 (0.37–1.44)	
5–10	9874	19	0.57 (0.34–0.91)	268	115	0.76 (0.59–0.96)	43	11	0.55 (0.26–1.10)	
10–20	19,742	44	0.67 (0.47–0.93)	494	273	0.98 (0.81–1.17)	80	27	0.72 (0.42–1.21)	
20–40	39,470	102	0.77 (0.60–1.00)	970	564	1.03 (0.89–1.19)	142	72	1.09 (0.73–1.63)	
40–60	39,440	132	1	979	564	1	146	68	1	
60–80	39,414	158	1.20 (0.95–1.51)	951	583	1.08 (0.94–1.25)	130	84	1.39 (0.93–2.07)	
80–90	19,697	88	1.33 (1.02–1.75)	456	311	1.21 (1.01–1.44)	61	46	1.62 (1.00–2.61)	
90–95	9853	41	1.24 (0.86–1.75)	236	147	1.10 (0.87–1.38)	35	19	1.17 (0.61–2.17)	
95–100	9834	60	1.82 (1.33–2.46)	220	164	1.32 (1.04–1.65)	27	27	2.15 (1.17–3.95)	
(d) Select and shrink										
0–5	9957	16	0.54 (0.31–0.89)	279	105	0.63 (0.49–0.81)	38	16	0.71 (0.36–1.33)	
5–10	9888	15	0.51 (0.29–0.85)	254	129	0.85 (0.67–1.08)	41	13	0.53 (0.26–1.03)	
10–20	19,812	51	0.87 (0.62–1.20)	489	278	0.96 (0.80–1.14)	81	26	0.54 (0.32–0.90)	
20–40	39,435	113	0.97 (0.75–1.25)	1013	521	0.86 (0.75–1.00)	156	58	0.62 (0.41–0.94)	
40–60	39,512	117	1	961	572	1	134	80	1	
60–80	39,316	158	1.36 (1.07–1.73)	950	584	1.03 (0.89–1.20)	137	77	0.94 (0.63–1.40)	
80–90	19,718	77	1.32 (0.98–1.76)	434	333	1.29 (1.08–1.54)	61	46	1.26 (0.79–2.02)	
90–95	9791	45	1.55 (1.09–2.17)	233	150	1.08 (0.86–1.36)	30	24	1.34 (0.73–2.45)	
95–100	9775	65	2.25 (1.65–3.03)	215	169	1.32 (1.05–1.66)	26	28	1.80 (0.99–3.31)	

*OR* odds ratio, *CI* confidence interval.

### Absolute risk of developing ovarian cancer by PRS percentiles

**Fig. 3:**
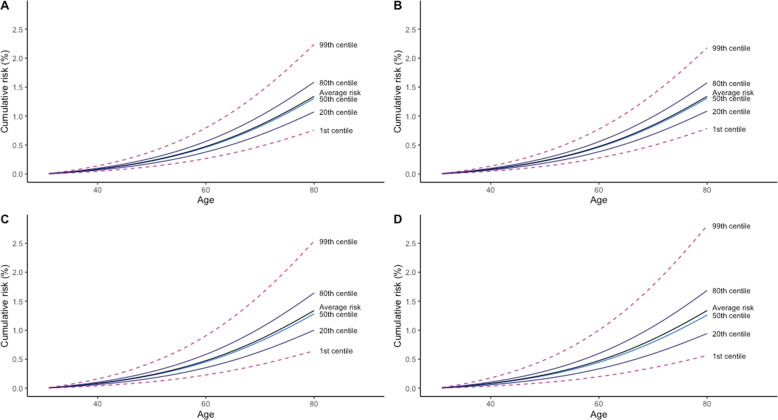
Cumulative risk of ovarian cancer between birth and age 80 by PRS percentiles and PRS models. Shown are the cumulative risk of ovarian cancer risk in UK women by polygenic risk score percentiles. The lasso (**A**) and elastic net (**B**) penalized regression models were applied to individual level genotype data, while the stepwise (**C**) and S4 (**D**) models were applied to summary level statistics. Note that the median and the mean risk differ because the distribution of the relative risk in the population is left-skewed (the log relative risk is a Normal distribution).

We estimated cumulative risk of EOC within PRS percentiles for women in the general population (Fig. 3), by applying the odds ratio from the PRS models to age-specific population incidence and mortality data for England in 2016. For *BRCA1* and *BRCA2* pathogenic variant carriers, we applied the estimated hazard ratios from PRS models to age-specific incidence rates obtained from Kuchenbaecker et al. [21]. For women in the general population, the estimated cumulative risks of EOC by age 80 for women at the 99th centile of the PRS distribution were 2.24%, 2.18%, 2.54%, and 2.81% for the lasso, elastic net, stepwise and S4 models, respectively. In comparison, the absolute risks of EOC by age 80 for women at the 1st centile were 0.76%, 0.78%, 0.64%, and 0.56% for the lasso, elastic net, stepwise and S4 models, respectively.

The absolute risks of developing EOC in *BRCA1* and *BRCA2* pathogenic variant carriers were considerably higher than for women in the general population (Figs. S1 and S2). The estimated absolute risk of developing ovarian cancer by age 80 for *BRCA1* carriers at the 99th PRS centiles were 63.2%, 66.3%, 59.0%, and 68.4% for the lasso, elastic net, stepwise and S4 models, respectively. The corresponding absolute risks for women at the 1st PRS centile were 27.7%, 25.6%, 30.8%, and 24.2%. For *BRCA2* carriers the absolute risks for women at the 99th centile were 36.3%, 36.3%, 33.0%, and 36.9%; and 7.10%, 7.12%, 8.24%, and 6.92% at the 1st centile for the lasso, elastic net, stepwise and S4 models, respectively.

### PRS distribution and ancestries

To investigate the transferability of the PRS to other populations, we applied the scores to women of African (*N* = 1,072) and Asian (*N* = 7,669) ancestries genotyped as part of the OncoArray project. In general, the distributions of the raw PRS were dependent on both the statistical methods used in SNP selection and ancestral group. PRS models that included more variants had less dispersion, such that the elastic net models had the least between individual variation in all ancestral groups (standard deviation = 0.15, 0.19, and 0.22 for individuals of Asian, African and European ancestries respectively), while the distributions from the stepwise models were the most dispersed (standard deviation = 0.23, 0.27, and 0.30 for individuals of Asian, African and European ancestries respectively). As expected, given the variation in variant frequencies by population, the distribution of polygenic scores was significantly different across the three ancestral groups, with the least dispersion among women of Asian ancestries and the most variation in women of European ancestries. The difference in PRS distribution was minimized after correction for ancestry by standardizing the PRS to have unit standard deviation using the control subjects for each ancestral group.

High PRSs were significantly associated with risk of non-mucinous EOC in both Asian and African ancestries (Table 4), although the effects were weaker than in women of European ancestries. For example, with the lasso model, the odds ratio per unit standard deviation increment in polygenic score was 1.16 (95% CI: 1.11–1.22) in women of East Asian ancestries, 1.28 (95% CI: 1.13–1.45) in women of African ancestries and 1.37 (95% CI: 1.27–1.48) in women of European ancestries (*p* for heterogeneity <0.0001). Variability in effect sizes among ancestral groups was highest for the stepwise model (*I*^2^ = 92%) versus 84% and 83% for elastic net and lasso derived polygenic scores respectively. The best discriminative model among women of East Asian and African ancestries were the elastic net PRS (AUC = 0.543) and the S4 PRS derived from OCAC-CIMBA meta-analysis (AUC = 0.596) respectively. Women of African ancestries in the top 5% of the PRS had about two-fold increased risk compared to women in the middle quintile (lasso OR: 1.64, 95% CI: 0.90–3.00; elastic net OR: 1.64, 95% CI: 0.90–3.00; stepwise OR: 2.15, 95% CI: 1.17–3.95; S4 OR: 1.80, 95% CI: 0.99–3.31) (Table 3). Effect estimates were smaller in women of East Asian ancestries with women in the top 5% of the PRS, having about a 1.5 fold increased risk compared to women in the middle quintile (lasso OR: 1.40, 95% CI: 1.12–1.76; elastic net OR: 1.60, 95% CI: 1.28–2.01; stepwise OR: 1.32, 95% CI: 1.04–1.65; S4 OR: 1.32, 95% CI: 1.05–1.66) (Table 3).

**Table 4:** External validation of PRS models in East Asian and African Populations.

Model	East Asian ancestries			African ancestries		
	AUC	OR	95% CI	AUC	OR	95% CI
Lasso	0.541	1.16	(1.11–1.22)	0.576	1.28	(1.13–1.45)
Elastic net	0.543	1.17	(1.12–1.23)	0.574	1.29	(1.14–1.47)
Stepwise (OCAC)	0.528	1.11	(1.06–1.16)	0.581	1.34	(1.18–1.52)
Select and shrink (OCAC)	0.538	1.14	(1.08–1.19)	0.593	1.38	(1.21–1.58)
Stepwise (OCAC-CIMBA)	0.542	1.17	(1.11–1.23)	0.594	1.37	(1.20–1.56)
Select and shrink (OCAC-CIMBA)	0.537	1.14	(1.08–1.19)	0.596	1.41	(1.23–1.61)

## Discussion

Genetic risk profiling with PRSs has led to actionable outcomes for cancers such as breast and prostate [22, 23]. Previous PRS scores for invasive EOC risk in the general population and *BRCA1/BRCA2* pathogenic variant carriers have been based on genetic variants for which an association with EOC risk had been established at nominal genome-wide significance [20, 24, 25]. Here, we explored the predictive performance of computationally efficient, penalized, regression methods in modeling joint SNP effects for EOC risk prediction in diverse populations and compared them with common approaches. By leveraging the correlation between SNPs which do not reach nominal genome-wide thresholds and including them in PRS models, the PRSs derived from penalized regression models provide stronger evidence of association with risk of non-mucinous EOC than previously published PRSs in both the general population and in *BRCA1/BRCA2* pathogenic variant carriers.

Recently, Barnes et al. derived a PRS score using 22 SNPs that were significantly associated with high-grade serous EOC risk (PRS_HGS_) to predict EOC risk in *BRCA1/BRCA2* pathogenic variant carriers [20]. To make effect estimates obtained in this analysis comparable to the effect estimates obtained from the PRS_HGS_, we standardized all PRSs using the standard deviation from unaffected *BRCA1/BRCA2* carriers and provide estimates which are directly comparable to the PRS_HGS_ in Supplementary Table 9. All PRS models in this analysis except the Stepwise (OCAC only) had higher effect estimates [20]. The AUC estimates from the adjusted PLR methods implemented in this analysis, are higher than the corresponding PRS_HGS_ estimates for *BRCA1* carriers (0.604). In *BRCA2* carriers, the AUC estimates for the lasso and S4 models did slightly better than the PRS_HGS_ AUC estimate (0.667), while the stepwise did slightly worse and the elastic net estimate was comparable. The AUC estimates for women in the general population, as estimated from the UK Biobank, are slightly higher than estimates from previously published PRS models for overall EOC risk by Jia et al. (AUC = 0.57) and Yang et al. (AUC = 0.58) [25, 26].

The level of risk for women above the 95th percentile of the PRS is similar to that conferred by pathogenic variants in moderate penetrance genes such as *FANCM* (RR = 2.1, 95% CI = 1.1–3.9) and *PALB2* (RR = 2.91 95% CI = 1.40–6.04) [27, 28]. The inclusion of other risk factors such as family history of ovarian cancer, presence of rare pathogenic variants, age at menarche, oral contraceptive use, hormone replacement therapy, parity, and endometriosis in combination with the PRS could potentially improve risk stratification as implemented in the CanRisk tool (www.canrisk.org), which currently uses a 36-SNP PRS with the potential to use other PRS models [29, 30].

We found that the discrimination of the PRS varied by ancestry with greater discrimination in women of European ancestries than in women of African and East Asian ancestries. The better performance in African than East Asian populations is in contrast to what one would expect given human demographic history, and the performance of PRS for other phenotypes in African populations. This may simply be the play of chance given the small number of samples of African ancestries. Alternatively it reflects the fact that the allele frequencies of the PRS SNPs were more similar between the African and European populations than they were with the East Asian population (Supplementary Tables 10–14).

Further optimization of the models could be achieved by varying the penalization function based on prior knowledge. For example, varying the penalty function to select more SNPs from genomic regions with known susceptibility variants given that susceptibility variants tend to cluster together. Alternatively, the penalty functions could be modified to incorporate information about functionally active regions of the genome such a promoters, enhancers, and transcription factor binding sites. However, incorporating functional annotation has resulted in limited gains in prediction accuracy for complex traits such as breast cancer, celiac disease, type 2 diabetes, and rheumatoid arthritis [31].

Machine/deep learning approaches are alternative ways to constructing PRS, but methods such as the neural net, support vector machine, and random forest have been shown to be computationally prohibitive or produce inferior results to other approaches [32, 33]. Other machine learning methods, such as those based on gradient boosting do not perform well in genomic regions where strong genetic interactions are present, for which alternative approaches such as the LDPred may perform better [18]. Our approach has several benefits over alternative machine learning methods, including its simplicity, and intrinsic robustness to minor misspecification of LD or association strength.

In conclusion, our results indicate that using the lasso model for individual level genotype data and the S4 model for summary level data in PRS construction provide an improvement in risk prediction for non-mucinous EOC over more common approaches. Our approach overcomes the computational limitations in the use of penalized methods for large-scale genetic data, particularly in the presence of highly correlated SNPs and when the use of cross-validation for parameter estimation is preferred. In practical terms, the PRS provides sufficient discrimination, particularly for women of European ancestries, to be considered for inclusion in risk prediction and prevention approaches for EOC in the future. Further studies are required to optimize these PRSs in ancestrally diverse populations and to validate their performance with the inclusion of other genetic and lifestyle risk factors.

## Supplementary information

Supplementary Material

FigureS1: Cumulative risk of ovarian cancer risk in BRCA1 carriers by polygenic risk score percentiles. The lasso (A) and elastic net (B) penalized regression models were applied to individual level g

Figure S2:Cumulative risk of ovarian cancer risk in BRCA2 carriers by polygenic risk score percentiles. The lasso (A) and elastic net (B) penalized regression models were applied to individual level g

Table S1- Lasso Weights

Table S2- Elastic Net Weights

Table S3- Stepwise Weights

Table S4-Select and Shrink OCAC Weights

Table S5-Stepwise OCAC CIMBA Weights

Table S6- Select and Shrink OCAC CIMBA Weights

Table S7- Hazard Ratios BRCA Carriers

Table S8-Absolute Risks BRCA Carriers 10th and 90th Percentile

Table S9-Adjusted and Unadjusted Models in BRCA Carriers

Table S10- Mean Allele Frequency Ancestries Lasso Model

Table S11 - Mean Allele Frequency Ancestries Elastic Net Model

Table S12 - Mean Allele Frequency Ancestries Stepwise Model

Table S13- Mean Allele Frequency Ancestries Select and Shrink OCAC Model

Table S14- Mean Allele Frequency Ancestries Select and Shrink OCAC CIMBA Model

## Data Availability

OncoArray germline genotype data for the OCAC studies have been deposited at the European Genome-phenome Archive (EGA; https://ega-archive.org/), which is hosted by the EBI and the CRG, under accession EGAS00001002305. Summary statisitics for the Ovarian Cancer Association Consortium are available in the NHGRI-EBI GWAS catalogue (https://www.ebi.ac.uk/gwas/home) under the accession number GCST90016665. A subset of the OncoArray germline genotype data for the CIMBA studies are publically available through the database of Genotypes and Phenotypes (dbGaP) under accession phs001321.v1.p1. The complete data set will not be made publically available because of restraints imposed by the ethics committees of individual studies; requests for further data can be made to the Data Access Coordination Committee (http://cimba.ccge.medschl.cam.ac.uk/)
