# Correction: Polygenic risk modeling for prediction of epithelial ovarian cancer risk

**DOI:** 10.1038/s41431-022-01085-y

**Published:** 2022-03-22

**Authors:** Eileen O. Dareng, Jonathan P. Tyrer, Daniel R. Barnes, Michelle R. Jones, Xin Yang, Katja K. H. Aben, Muriel A. Adank, Simona Agata, Irene L. Andrulis, Hoda Anton-Culver, Natalia N. Antonenkova, Gerasimos Aravantinos, Banu K. Arun, Annelie Augustinsson, Judith Balmaña, Elisa V. Bandera, Rosa B. Barkardottir, Daniel Barrowdale, Matthias W. Beckmann, Alicia Beeghly-Fadiel, Javier Benitez, Marina Bermisheva, Marcus Q. Bernardini, Line Bjorge, Amanda Black, Natalia V. Bogdanova, Bernardo Bonanni, Ake Borg, James D. Brenton, Agnieszka Budzilowska, Ralf Butzow, Saundra S. Buys, Hui Cai, Maria A. Caligo, Ian Campbell, Rikki Cannioto, Hayley Cassingham, Jenny Chang-Claude, Stephen J. Chanock, Kexin Chen, Yoke-Eng Chiew, Wendy K. Chung, Kathleen B. M. Claes, Sarah Colonna, GEMO Study Collaborators, Fabienne Lesueur, Noura Mebirouk, GC-HBOC Study Collaborators, Christoph Engel, Rita K. Schmutzler, EMBRACE Collaborators, Daniel Barrowdale, Eleanor Davies, Diana M. Eccles, D. Gareth Evans, Linda S. Cook, Fergus J. Couch, Mary B. Daly, Fanny Dao, Eleanor Davies, Miguel de la Hoya, Robin de Putter, Joe Dennis, Allison DePersia, Peter Devilee, Orland Diez, Yuan Chun Ding, Jennifer A. Doherty, Susan M. Domchek, Thilo Dörk, Andreas du Bois, Matthias Dürst, Diana M. Eccles, Heather A. Eliassen, Christoph Engel, Gareth D. Evans, Peter A. Fasching, James M. Flanagan, Renée T. Fortner, Eva Machackova, Eitan Friedman, Patricia A. Ganz, Judy Garber, Francesca Gensini, Graham G. Giles, Gord Glendon, Andrew K. Godwin, Marc T. Goodman, Mark H. Greene, Jacek Gronwald, OPAL Study Group, AOCS Group, Eric Hahnen, Christopher A. Haiman, Niclas Håkansson, Ute Hamann, Thomas V. O. Hansen, Holly R. Harris, Mikael Hartman, Florian Heitz, Michelle A. T. Hildebrandt, Estrid Høgdall, Claus K. Høgdall, John L. Hopper, Ruea-Yea Huang, Chad Huff, Peter J. Hulick, David G. Huntsman, Evgeny N. Imyanitov, KConFab Investigators, Georgia Chenevix-Trench, HEBON Investigators, Muriel A. Adank, Peter Devilee, Annemieke H. van der Hout, Claudine Isaacs, Anna Jakubowska, Paul A. James, Ramunas Janavicius, Allan Jensen, Oskar Th. Johannsson, Esther M. John, Michael E. Jones, Daehee Kang, Beth Y. Karlan, Anthony Karnezis, Linda E. Kelemen, Elza Khusnutdinova, Lambertus A. Kiemeney, Byoung-Gie Kim, Susanne K. Kjaer, Ian Komenaka, Jolanta Kupryjanczyk, Allison W. Kurian, Ava Kwong, Diether Lambrechts, Melissa C. Larson, Conxi Lazaro, Nhu D. Le, Goska Leslie, Jenny Lester, Fabienne Lesueur, Douglas A. Levine, Lian Li, Jingmei Li, Jennifer T. Loud, Karen H. Lu, Jan Lubiński, Phuong L. Mai, Siranoush Manoukian, Jeffrey R. Marks, Rayna Kim Matsuno, Keitaro Matsuo, Taymaa May, Lesley McGuffog, John R. McLaughlin, Iain A. McNeish, Noura Mebirouk, Usha Menon, Austin Miller, Roger L. Milne, Albina Minlikeeva, Francesmary Modugno, Marco Montagna, Kirsten B. Moysich, Elizabeth Munro, Katherine L. Nathanson, Susan L. Neuhausen, Heli Nevanlinna, Joanne Ngeow Yuen Yie, Henriette Roed Nielsen, Finn C. Nielsen, Liene Nikitina-Zake, Kunle Odunsi, Kenneth Offit, Edith Olah, Siel Olbrecht, Olufunmilayo I. Olopade, Sara H. Olson, Håkan Olsson, Ana Osorio, Laura Papi, Sue K. Park, Michael T. Parsons, Harsha Pathak, Inge Sokilde Pedersen, Ana Peixoto, Tanja Pejovic, Pedro Perez-Segura, Jennifer B. Permuth, Beth Peshkin, Paolo Peterlongo, Anna Piskorz, Darya Prokofyeva, Paolo Radice, Johanna Rantala, Marjorie J. Riggan, Harvey A. Risch, Cristina Rodriguez-Antona, Eric Ross, Mary Anne Rossing, Ingo Runnebaum, Dale P. Sandler, Marta Santamariña, Penny Soucy, Rita K. Schmutzler, V. Wendy Setiawan, Kang Shan, Weiva Sieh, Jacques Simard, Christian F. Singer, Anna P. Sokolenko, Honglin Song, Melissa C. Southey, Helen Steed, Dominique Stoppa-Lyonnet, Rebecca Sutphen, Anthony J. Swerdlow, Yen Yen Tan, Manuel R. Teixeira, Soo Hwang Teo, Kathryn L. Terry, Mary Beth Terry, The OCAC Consortium, Eileen O. Dareng, Jonathan P. Tyrer, Michelle R. Jones, Katja K. H. Aben, Hoda Anton-Culver, Natalia N. Antonenkova, Gerasimos Aravantinos, Matthias W. Beckmann, Alicia Beeghly-Fadiel, Javier Benitez, Marina Bermisheva, Marcus Q. Bernardini, Line Bjorge, Natalia V. Bogdanova, James D. Brenton, Agnieszka Budzilowska, Ralf Butzow, Hui Cai, Ian Campbell, Rikki Cannioto, Jenny Chang-Claude, Stephen J. Chanock, Kexin Chen, Yoke-Eng Chiew, Linda S. Cook, Fanny Dao, Joe Dennis, Jennifer A. Doherty, Thilo Dörk, Andreas du Bois, Matthias Dürst, Diana M. Eccles, Heather A. Eliassen, Peter A. Fasching, James M. Flanagan, Renée T. Fortner, Graham G. Giles, Marc T. Goodman, Jacek Gronwald, Christopher A. Haiman, Niclas Håkansson, Holly R. Harris, Florian Heitz, Michelle A. T. Hildebrandt, Estrid Høgdall, Claus K. Høgdall, Ruea-Yea Huang, Chad Huff, David G. Huntsman, Anna Jakubowska, Allan Jensen, Michael E. Jones, Daehee Kang, Beth Y. Karlan, Anthony Karnezis, Linda E. Kelemen, Elza Khusnutdinova, Lambertus A. Kiemeney, Byoung-Gie Kim, Susanne K. Kjaer, Jolanta Kupryjanczyk, Diether Lambrechts, Melissa C. Larson, Nhu D. Le, Jenny Lester, Douglas A. Levine, Karen H. Lu, Jan Lubiński, Jeffrey R. Marks, Rayna Kim Matsuno, Keitaro Matsuo, Taymaa May, John R. McLaughlin, Iain A. McNeish, Roger L. Milne, Albina Minlikeeva, Francesmary Modugno, Kirsten B. Moysich, Elizabeth Munro, Heli Nevanlinna, Kunle Odunsi, Siel Olbrecht, Sara H. Olson, Håkan Olsson, Ana Osorio, Sue K. Park, Tanja Pejovic, Jennifer B. Permuth, Anna Piskorz, Darya Prokofyeva, Marjorie J. Riggan, Harvey A. Risch, Cristina Rodriguez-Antona, Mary Anne Rossing, Ingo Runnebaum, Dale P. Sandler, V. Wendy Setiawan, Kang Shan, Weiva Sieh, Honglin Song, Melissa C. Southey, Helen Steed, Rebecca Sutphen, Anthony J. Swerdlow, Soo Hwang Teo, Kathryn L. Terry, Pamela J. Thompson, Liv Cecilie Vestrheim Thomsen, Linda Titus, Britton Trabert, Ruth Travis, Shelley S. Tworoger, Ellen Valen, Anne M. van Altena, Els Van Nieuwenhuysen, Digna Velez Edwards, Robert A. Vierkant, Frances Wang, Penelope M. Webb, Clarice R. Weinberg, Nicolas Wentzensen, Emily White, Alice S. Whittemore, Stacey J. Winham, Alicja Wolk, Yin-Ling Woo, Anna H. Wu, Li Yan, Drakoulis Yannoukakos, Wei Zheng, Argyrios Ziogas, Kate Lawrenson, Anna deFazio, Susan J. Ramus, Celeste L. Pearce, Alvaro N. Monteiro, Julie M. Cunningham, Ellen L. Goode, Joellen M. Schildkraut, Andrew Berchuck, Simon A. Gayther, Paul D. P. Pharoah, The CIMBA Consortium, Daniel R. Barnes, Xin Yang, Muriel A. Adank, Simona Agata, Irene L. Andrulis, Banu K. Arun, Annelie Augustinsson, Judith Balmaña, Rosa B. Barkardottir, Daniel Barrowdale, Bernardo Bonanni, Ake Borg, Saundra S. Buys, Maria A. Caligo, Hayley Cassingham, Wendy K. Chung, Kathleen B. M. Claes, Sarah Colonna, Fergus J. Couch, Mary B. Daly, Eleanor Davies, Miguel de la Hoya, Robin de Putter, Allison DePersia, Peter Devilee, Orland Diez, Yuan Chun Ding, Susan M. Domchek, Diana M. Eccles, Christoph Engel, D. Gareth Evans, Eva Machackova, Eitan Friedman, Patricia A. Ganz, Judy Garber, Francesca Gensini, Gord Glendon, Andrew K. Godwin, Mark H. Greene, Eric Hahnen, Ute Hamann, Thomas V. O. Hansen, Mikael Hartman, John L. Hopper, Peter J. Hulick, Evgeny N. Imyanitov, Claudine Isaacs, Paul A. James, Ramunas Janavicius, Oskar Th. Johannsson, Esther M. John, Ian Komenaka, Allison W. Kurian, Ava Kwong, Conxi Lazaro, Goska Leslie, Fabienne Lesueur, Jingmei Li, Jennifer T. Loud, Phuong L. Mai, Siranoush Manoukian, Lesley McGuffog, Noura Mebirouk, Austin Miller, Marco Montagna, Katherine L. Nathanson, Susan L. Neuhausen, Joanne Ngeow Yuen Yie, Henriette Roed Nielsen, Liene Nikitina-Zake, Kenneth Offit, Edith Olah, Olufunmilayo I. Olopade, Laura Papi, Michael T. Parsons, Harsha Pathak, Inge Sokilde Pedersen, Ana Peixoto, Pedro Perez-Segura, Beth Peshkin, Paolo Peterlongo, Paolo Radice, Johanna Rantala, Eric Ross, Marta Santamariña, Penny Soucy, Rita K. Schmutzler, Jacques Simard, Christian F. Singer, Anna P. Sokolenko, Dominique Stoppa-Lyonnet, Yen Yen Tan, Manuel R. Teixeira, Mary Beth Terry, Mads Thomassen, Darcy L. Thull, Marc Tischkowitz, Amanda E. Toland, Diana Torres, Nadine Tung, Annemieke H. van der Hout, Elizabeth J. van Rensburg, Ana Vega, Barbara Wappenschmidt, Jeffrey N. Weitzel, Katia M. Zavaglia, Kristin K. Zorn, Thomas A. Sellers, Georgia Chenevix-Trench, Antonis C. Antoniou, Mads Thomassen, Pamela J. Thompson, Liv Cecilie Vestrheim Thomsen, Darcy L. Thull, Marc Tischkowitz, Linda Titus, Amanda E. Toland, Diana Torres, Britton Trabert, Ruth Travis, Nadine Tung, Shelley S. Tworoger, Ellen Valen, Anne M. van Altena, Annemieke H. van der Hout, Els Van Nieuwenhuysen, Elizabeth J. van Rensburg, Ana Vega, Digna Velez Edwards, Robert A. Vierkant, Frances Wang, Barbara Wappenschmidt, Penelope M. Webb, Clarice R. Weinberg, Jeffrey N. Weitzel, Nicolas Wentzensen, Emily White, Alice S. Whittemore, Stacey J. Winham, Alicja Wolk, Yin-Ling Woo, Anna H. Wu, Li Yan, Drakoulis Yannoukakos, Katia M. Zavaglia, Wei Zheng, Argyrios Ziogas, Kristin K. Zorn, Zdenek Kleibl, Douglas Easton, Kate Lawrenson, Anna DeFazio, Thomas A. Sellers, Susan J. Ramus, Celeste L. Pearce, Alvaro N. Monteiro, Julie Cunningham, Ellen L. Goode, Joellen M. Schildkraut, Andrew Berchuck, Georgia Chenevix-Trench, Simon A. Gayther, Antonis C. Antoniou, Paul D. P. Pharoah

**Affiliations:** 1 University of Cambridge, Centre for Cancer Genetic Epidemiology, Department of Public Health and Primary Care, Cambridge, UK; 2 University of Cambridge, Centre for Cancer Genetic Epidemiology, Department of Oncology, Cambridge, UK; 3 Center for Bioinformatics and Functional Genomics, Cedars-Sinai Medical Center, Los Angeles, CA USA; 4 Radboud University Medical Center, Radboud Institute for Health Sciences, Nijmegen, The Netherlands; 5 Netherlands Comprehensive Cancer Organisation, Utrecht, The Netherlands; 6 The Netherlands Cancer Institute—Antoni van Leeuwenhoek hospital, Family Cancer Clinic, Amsterdam, The Netherlands; 7 Veneto Institute of Oncology IOV—IRCCS, Immunology and Molecular Oncology Unit, Padua, Italy; 8 Lunenfeld-Tanenbaum Research Institute of Mount Sinai Hospital, Fred A. Litwin Center for Cancer Genetics, Toronto, ON Canada; 9 University of Toronto, Department of Molecular Genetics, Toronto, ON Canada; 10 University of California Irvine, Department of Epidemiology, Genetic Epidemiology Research Institute, Irvine, CA USA; 11 N.N. Alexandrov Research Institute of Oncology and Medical Radiology, Minsk, Belarus; 12 ‘Agii Anargiri’ Cancer Hospital, Athens, Greece; 13 University of Texas MD Anderson Cancer Center, Department of Breast Medical Oncology, Houston, TX USA; 14 Lund University, Department of Cancer Epidemiology, Clinical Sciences, Lund, Sweden; 15 Vall d’Hebron Institute of Oncology, Hereditary cancer Genetics Group, Barcelona, Spain; 16 University Hospital of Vall d’Hebron, Department of Medical Oncology, Barcelona, Spain; 17 Rutgers Cancer Institute of New Jersey, Cancer Prevention and Control Program, New Brunswick, NJ USA; 18 Landspitali University Hospital, Department of Pathology, Reykjavik, Iceland; 19 University of Iceland, BMC (Biomedical Centre), Faculty of Medicine, Reykjavik, Iceland; 20 University Hospital Erlangen, Friedrich-Alexander-University Erlangen-Nuremberg, Department of Gynecology and Obstetrics, Comprehensive Cancer Center ER-EMN, Erlangen, Germany; 21 Vanderbilt University School of Medicine, Division of Epidemiology, Department of Medicine, Vanderbilt Epidemiology Center, Vanderbilt-Ingram Cancer Center, Nashville, TN USA; 22 Biomedical Network on Rare Diseases (CIBERER), Madrid, Spain; 23 Spanish National Cancer Research Centre (CNIO), Human Cancer Genetics Programme, Madrid, Spain; 24 Ufa Federal Research Centre of the Russian Academy of Sciences, Institute of Biochemistry and Genetics, Ufa, Russia; 25 Princess Margaret Hospital, Division of Gynecologic Oncology, University Health Network, Toronto, ON Canada; 26 Haukeland University Hospital, Department of Obstetrics and Gynecology, Bergen, Norway; 27 University of Bergen, Centre for Cancer Biomarkers CCBIO, Department of Clinical Science, Bergen, Norway; 28 National Cancer Institute, Division of Cancer Epidemiology and Genetics, Bethesda, MD USA; 29 Hannover Medical School, Department of Radiation Oncology, Hannover, Germany; 30 Hannover Medical School, Gynaecology Research Unit, Hannover, Germany; 31 IEO, European Institute of Oncology IRCCS, Division of Cancer Prevention and Genetics, Milan, Italy; 32 Lund University and Skåne University Hospital, Department of Oncology, Lund, Sweden; 33 Cancer Research UK Cambridge Institute, University of Cambridge, Cambridge, UK; 34 Maria Sklodowska-Curie National Research Institute of Oncology, Department of Pathology and Laboratory Diagnostics, Warsaw, Poland; 35 University of Helsinki, Department of Pathology, Helsinki University Hospital, Helsinki, Finland; 36 Huntsman Cancer Institute, Department of Medicine, Salt Lake City, UT USA; 37 University Hospital, SOD Genetica Molecolare, Pisa, Italy; 38 Peter MacCallum Cancer Center, Melbourne, VIC Australia; 39 The University of Melbourne, Sir Peter MacCallum Department of Oncology, Melbourne, VIC Australia; 40 Roswell Park Cancer Institute, Cancer Pathology & Prevention, Division of Cancer Prevention and Population Sciences, Buffalo, NY USA; 41 Division of Human Genetics, The Ohio State University, Department of Internal Medicine, Columbus, OH USA; 42 German Cancer Research Center (DKFZ), Division of Cancer Epidemiology, Heidelberg, Germany; 43 University Medical Center Hamburg-Eppendorf, Cancer Epidemiology Group, University Cancer Center Hamburg (UCCH), Hamburg, Germany; 44 National Cancer Institute, National Institutes of Health, Department of Health and Human Services, Division of Cancer Epidemiology and Genetics, Bethesda, MD USA; 45 Tianjin Medical University Cancer Institute and Hospital, Department of Epidemiology, Tianjin, China; 46 The University of Sydney, Centre for Cancer Research, The Westmead Institute for Medical Research, Sydney, NSW Australia; 47 Westmead Hospital, Department of Gynaecological Oncology, Sydney, NSW Australia; 48 Columbia University, Departments of Pediatrics and Medicine, New York, NY USA; 49 Ghent University, Centre for Medical Genetics, Gent, Belgium; 50 INSERM U830, Department of Tumour Biology, Paris, France; 51 Institut Curie, Paris, France; 52 Mines ParisTech, Fontainebleau, France; 53 Faculty of Medicine and University Hospital Cologne, University of Cologne, Center for Familial Breast and Ovarian Cancer, Cologne, Germany; 54 University of New Mexico, University of New Mexico Health Sciences Center, Albuquerque, NM USA; 55 Alberta Health Services, Department of Cancer Epidemiology and Prevention Research, Calgary, AB Canada; 56 Mayo Clinic, Department of Laboratory Medicine and Pathology, Rochester, MN USA; 57 Fox Chase Cancer Center, Department of Clinical Genetics, Philadelphia, PA USA; 58 Memorial Sloan Kettering Cancer Center, Gynecology Service, Department of Surgery, New York, NY USA; 59 Cambridge, Cambridge, UK; 60 CIBERONC, Hospital Clinico San Carlos, IdISSC (Instituto de Investigación Sanitaria del Hospital Clínico San Carlos), Molecular Oncology Laboratory, Madrid, Spain; 61 NorthShore University Health System, Center for Medical Genetics, Evanston, IL USA; 62 The University of Chicago Pritzker School of Medicine, Chicago, IL USA; 63 Leiden University Medical Center, Department of Pathology, Leiden, The Netherlands; 64 Leiden University Medical Center, Department of Human Genetics, Leiden, The Netherlands; 65 Vall dHebron Institute of Oncology (VHIO), Oncogenetics Group, Barcelona, Spain; 66 University Hospital Vall dHebron, Clinical and Molecular Genetics Area, Barcelona, Spain; 67 Beckman Research Institute of City of Hope, Department of Population Sciences, Duarte, CA USA; 68 University of Utah, Huntsman Cancer Institute, Department of Population Health Sciences, Salt Lake City, UT USA; 69 University of Pennsylvania, Basser Center for BRCA, Abramson Cancer Center, Philadelphia, PA USA; 70 Ev. Kliniken Essen-Mitte (KEM), Department of Gynecology and Gynecologic Oncology, Essen, Germany; 71 Dr. Horst Schmidt Kliniken Wiesbaden, Department of Gynecology and Gynecologic Oncology, Wiesbaden, Germany; 72 Jena University Hospital—Friedrich Schiller University, Department of Gynaecology, Jena, Germany; 73 University of Southampton, Faculty of Medicine, Southampton, UK; 74 Harvard T.H. Chan School of Public Health, Department of Epidemiology, Boston, MA USA; 75 Brigham and Women’s Hospital and Harvard Medical School, Channing Division of Network Medicine, Boston, MA USA; 76 University of Leipzig, Institute for Medical Informatics, Statistics and Epidemiology, Leipzig, Germany; 77 University of Leipzig, LIFE—Leipzig Research Centre for Civilization Diseases, Leipzig, Germany; 78 University of Manchester, Manchester Academic Health Science Centre, Division of Evolution and Genomic Sciences, School of Biological Sciences, Faculty of Biology, Medicine and Health, Manchester, UK; 79 St Mary’s Hospital, Manchester University NHS Foundation Trust, Manchester Academic Health Science Centre, North West Genomics Laboratory Hub, Manchester Centre for Genomic Medicine, Manchester, UK; 80 University of California at Los Angeles, David Geffen School of Medicine, Department of Medicine Division of Hematology and Oncology, Los Angeles, CA USA; 81 Imperial College London, Division of Cancer and Ovarian Cancer Action Research Centre, Department of Surgery and Cancer, London, UK; 82 Masaryk Memorial Cancer Institute, Department of Cancer Epidemiology and Genetics, Brno, Czech Republic; 83 Chaim Sheba Medical Center, The Susanne Levy Gertner Oncogenetics Unit, Ramat Gan, Israel; 84 Tel Aviv University, Sackler Faculty of Medicine, Ramat Aviv, Israel; 85 Jonsson Comprehensive Cancer Centre, UCLA, Schools of Medicine and Public Health, Division of Cancer Prevention & Control Research, Los Angeles, CA USA; 86 Dana-Farber Cancer Institute, Cancer Risk and Prevention Clinic, Boston, MA USA; 87 University of Florence, Department of Experimental and Clinical Biomedical Sciences ‘Mario Serio’, Medical Genetics Unit, Florence, Italy; 88 Cancer Council Victoria, Cancer Epidemiology Division, Melbourne, VIC Australia; 89 The University of Melbourne, Centre for Epidemiology and Biostatistics, Melbourne School of Population and Global Health, Melbourne, VIC Australia; 90 Monash University, Precision Medicine, School of Clinical Sciences at Monash Health, Clayton, VIC Australia; 91 University of Kansas Medical Center, Department of Pathology and Laboratory Medicine, Kansas City, KS USA; 92 Cedars-Sinai Medical Center, Samuel Oschin Comprehensive Cancer Institute, Cancer Prevention and Genetics Program, Los Angeles, CA USA; 93 National Cancer Institute, Clinical Genetics Branch, Division of Cancer Epidemiology and Genetics, Bethesda, MD USA; 94 Pomeranian Medical University, Department of Genetics and Pathology, Szczecin, Poland; 95 QIMR Berghofer Medical Research Institute, Population Health Department, Brisbane, QLD Australia; 96 Faculty of Medicine and University Hospital Cologne, University of Cologne, Center for Integrated Oncology (CIO), Cologne, Germany; 97 University of Southern California, Department of Preventive Medicine, Keck School of Medicine, Los Angeles, CA USA; 98 Karolinska Institutet, Institute of Environmental Medicine, Stockholm, Sweden; 99 German Cancer Research Center (DKFZ), Molecular Genetics of Breast Cancer, Heidelberg, Germany; 100 Rigshospitalet, Copenhagen University Hospital, Department of Clinical Genetics, Copenhagen, Denmark; 101 Fred Hutchinson Cancer Research Center, Program in Epidemiology, Division of Public Health Sciences, Seattle, WA USA; 102 University of Washington, Department of Epidemiology, Seattle, WA USA; 103 National University of Singapore and National University Health System, Saw Swee Hock School of Public Health, Singapore, Singapore; 104 National University Health System, Department of Surgery, Singapore, Singapore; 105 Humboldt-Universität zu Berlin, and Berlin Institute of Health, Department for Gynecology with the Center for Oncologic Surgery Charité Campus Virchow-Klinikum, Charité – Universitätsmedizin Berlin, corporate member of Freie Universität Berlin, Berlin, Germany; 106 University of Texas MD Anderson Cancer Center, Department of Epidemiology, Houston, TX USA; 107 Danish Cancer Society Research Center, Department of Virus, Lifestyle and Genes, Copenhagen, Denmark; 108 University of Copenhagen, Molecular Unit, Department of Pathology, Herlev Hospital, Copenhagen, Denmark; 109 University of Copenhagen, Department of Gynaecology, Rigshospitalet, Copenhagen Denmark; 110 Roswell Park Cancer Institute, Center For Immunotherapy, Buffalo, NY USA; 111 BC Cancer, Vancouver General Hospital, and University of British Columbia, British Columbia’s Ovarian Cancer Research (OVCARE) Program, Vancouver, BC Canada; 112 University of British Columbia, Department of Pathology and Laboratory Medicine, Vancouver, BC Canada; 113 University of British Columbia, Department of Obstetrics and Gynecology, Vancouver, BC Canada; 114 BC Cancer Research Centre, Department of Molecular Oncology, Vancouver, BC Canada; 115 N.N. Petrov Institute of Oncology, St. Petersburg, Russia; 116 Coordinating center: The Netherlands Cancer Institute, The Hereditary Breast and Ovarian Cancer Research Group Netherlands (HEBON), Amsterdam, The Netherlands; 117 Lombardi Comprehensive Cancer Center, Georgetown University, Washington, DC USA; 118 Pomeranian Medical University, Independent Laboratory of Molecular Biology and Genetic Diagnostics, Szczecin, Poland; 119 Peter MacCallum Cancer Center, Parkville Familial Cancer Centre, Melbourne, VIC Australia; 120 Vilnius University Hospital Santariskiu Clinics, Hematology, oncology and transfusion medicine center, Dept. of Molecular and Regenerative Medicine, Vilnius, Lithuania; 121 State Research Institute Centre for Innovative Medicine, Vilnius, Lithuania; 122 Landspitali University Hospital, Department of Oncology, Reykjavik, Iceland; 123 Stanford University School of Medicine, Department of Epidemiology & Population Health, Stanford, CA USA; 124 Stanford Cancer Institute, Stanford University School of Medicine, Department of Medicine, Division of Oncology, Stanford, CA USA; 125 The Institute of Cancer Research, Division of Genetics and Epidemiology, London, UK; 126 Seoul National University College of Medicine, Department of Preventive Medicine, Seoul, Korea; 127 Seoul National University Graduate School, Department of Biomedical Sciences, Seoul, Korea; 128 Seoul National University, Cancer Research Institute, Seoul, Korea; 129 University of California at Los Angeles, David Geffen School of Medicine, Department of Obstetrics and Gynecology, Los Angeles, CA USA; 130 UC Davis Medical Center, Department of Pathology and Laboratory Medicine, Sacramento, CA USA; 131 Medical University of South Carolina, Hollings Cancer Center, Charleston, SC USA; 132 Saint Petersburg State University, Saint Petersburg, Russia; 133 Sungkyunkwan University School of Medicine, Department of Obstetrics and Gynecology, Samsung Medical Center, Seoul, Korea; 134 City of Hope Clinical Cancer Genetics Community Research Network, Duarte, CA USA; 135 Cancer Genetics Centre, Hong Kong Hereditary Breast Cancer Family Registry, Happy Valley, Hong Kong; 136 The University of Hong Kong, Department of Surgery, Pok Fu Lam, Hong Kong; 137 Hong Kong Sanatorium and Hospital, Department of Surgery, Happy Valley, Hong Kong; 138 VIB Center for Cancer Biology, Leuven, Belgium; 139 University of Leuven, Laboratory for Translational Genetics, Department of Human Genetics, Leuven, Belgium; 140 Mayo Clinic, Department of Health Sciences Research, Division of Biomedical Statistics and Informatics, Rochester, MN USA; 141 ONCOBELL-IDIBELL-IGTP, Catalan Institute of Oncology, CIBERONC, Hereditary Cancer Program, Barcelona, Spain; 142 BC Cancer, Cancer Control Research, Vancouver, BC Canada; 143 Inserm U900, Genetic Epidemiology of Cancer team, Paris, France; 144 NYU Langone Medical Center, Gynecologic Oncology, Laura and Isaac Pearlmutter Cancer Center, New York, NY USA; 145 Genome Institute of Singapore, Human Genetics Division, Singapore, Singapore; 146 University of Texas MD Anderson Cancer Center, Department of Gynecologic Oncology and Clinical Cancer Genetics Program, Houston, TX USA; 147 Magee-Womens Hospital, University of Pittsburgh School of Medicine, Pittsburgh, PA USA; 148 Fondazione IRCCS Istituto Nazionale dei Tumori di Milano, Unit of Medical Genetics, Department of Medical Oncology and Hematology, Milan, Italy; 149 Duke University Hospital, Department of Surgery, Durham, NC USA; 150 University of Hawaii Cancer Center, Cancer Epidemiology Program, Honolulu, HI USA; 151 Aichi Cancer Center Research Institute, Division of Cancer Epidemiology and Prevention, Nagoya, Japan; 152 Nagoya University Graduate School of Medicine, Division of Cancer Epidemiology, Nagoya, Japan; 153 Samuel Lunenfeld Research Institute, Public Health Ontario, Toronto, ON Canada; 154 Imperial College London, Division of Cancer and Ovarian Cancer Action Research Centre, Department Surgery & Cancer, London, UK; 155 University of Glasgow, Institute of Cancer Sciences, Glasgow, UK; 156 University College London, MRC Clinical Trials Unit at UCL, Institute of Clinical Trials & Methodology, London, UK; 157 Roswell Park Cancer Institute, NRG Oncology, Statistics and Data Management Center, Buffalo, NY USA; 158 Roswell Park Cancer Institute, Division of Cancer Prevention and Control, Buffalo, NY USA; 159 Magee-Womens Research Institute and Hillman Cancer Center, Womens Cancer Research Center, Pittsburgh, PA USA; 160 University of Pittsburgh School of Medicine, Division of Gynecologic Oncology, Department of Obstetrics, Gynecology and Reproductive Sciences, Pittsburgh, PA USA; 161 Oregon Health & Science University, Department of Obstetrics and Gynecology, Portland, OR USA; 162 Oregon Health & Science University, Knight Cancer Institute, Portland, OR USA; 163 University of Helsinki, Department of Obstetrics and Gynecology, Helsinki University Hospital, Helsinki, Finland; 164 National Cancer Centre, Cancer Genetics Service, Singapore, Singapore; 165 Nanyang Technological University, Lee Kong Chian School of Medicine, Singapore, Singapore; 166 Odense University Hospital, Department of Clinical Genetics, Odence C, Denmark; 167 Latvian Biomedical Research and Study Centre, Riga, Latvia; 168 Roswell Park Cancer Institute, Department of Gynecologic Oncology, Buffalo, NY USA; 169 Memorial Sloan Kettering Cancer Center, Clinical Genetics Research Lab, Department of Cancer Biology and Genetics, New York, NY USA; 170 Memorial Sloan Kettering Cancer Center, Clinical Genetics Service, Department of Medicine, New York, NY USA; 171 National Institute of Oncology, Department of Molecular Genetics, Budapest, Hungary; 172 University Hospitals Leuven, Division of Gynecologic Oncology, Department of Obstetrics and Gynaecology and Leuven Cancer Institute, Leuven, Belgium; 173 The University of Chicago, Center for Clinical Cancer Genetics, Chicago, IL USA; 174 Memorial Sloan-Kettering Cancer Center, Department of Epidemiology and Biostatistics, New York, NY USA; 175 Centro de Investigación en Red de Enfermedades Raras (CIBERER), Madrid, Spain; 176 QIMR Berghofer Medical Research Institute, Department of Genetics and Computational Biology, Brisbane, QLD Australia; 177 Aalborg University Hospital, Molecular Diagnostics, Aalborg, Denmark; 178 Aalborg University Hospital, Clinical Cancer Research Center, Aalborg, Denmark; 179 Aalborg University, Department of Clinical Medicine, Aalborg, Denmark; 180 Portuguese Oncology Institute, Department of Genetics, Porto, Portugal; 181 Moffitt Cancer Center, Department of Cancer Epidemiology, Tampa, FL USA; 182 IFOM—the FIRC Institute of Molecular Oncology, Genome Diagnostics Program, Milan, Italy; 183 Bashkir State University, Department of Genetics and Fundamental Medicine, Ufa, Russia; 184 Fondazione IRCCS Istituto Nazionale dei Tumori (INT), Unit of Molecular Bases of Genetic Risk and Genetic Testing, Department of Research, Milan, Italy; 185 Karolinska Institutet, Clinical Genetics, Stockholm, Sweden; 186 Duke University Hospital, Department of Gynecologic Oncology, Durham, NC USA; 187 Yale School of Public Health, Chronic Disease Epidemiology, New Haven, CT USA; 188 Fox Chase Cancer Center, Population Studies Facility, Philadelphia, PA USA; 189 National Institute of Environmental Health Sciences, NIH, Epidemiology Branch, Research Triangle Park, NC USA; 190 Fundación Pública Galega Medicina Xenómica, Santiago De Compostela, Spain; 191 Instituto de Investigación Sanitaria de Santiago de Compostela, Santiago De Compostela, Spain; 192 Centre Hospitalier Universitaire de Québec – Université Laval Research Center, Genomics Center, Québec City, QC Canada; 193 Faculty of Medicine and University Hospital Cologne, University of Cologne, Center for Molecular Medicine Cologne (CMMC), Cologne, Germany; 194 Hebei Medical University, Fourth Hospital, Department of Obstetrics and Gynaecology, Shijiazhuang, China; 195 Icahn School of Medicine at Mount Sinai, Department of Population Health Science and Policy, New York, NY USA; 196 Icahn School of Medicine at Mount Sinai, Department of Genetics and Genomic Sciences, New York, NY USA; 197 Centre Hospitalier Universitaire de Québec-Université Laval Research Center, Genomic Center, Québec City, QC Canada; 198 Medical University of Vienna, Dept of OB/GYN and Comprehensive Cancer Center, Vienna, Austria; 199 University of Cambridge, Department of Public Health and Primary Care, Cambridge, UK; 200 The University of Melbourne, Department of Clinical Pathology, Melbourne, VIC Australia; 201 Royal Alexandra Hospital, Department of Obstetrics and Gynecology, Division of Gynecologic Oncology, Edmonton, AB Canada; 202 Institut Curie, Service de Génétique, Paris, France; 203 Université Paris Descartes, Paris, France; 204 University of South Florida, Epidemiology Center, College of Medicine, Tampa, FL USA; 205 The Institute of Cancer Research, Division of Breast Cancer Research, London, UK; 206 University of Porto, Biomedical Sciences Institute (ICBAS), Porto, Portugal; 207 Cancer Research Malaysia, Breast Cancer Research Programme, Subang Jaya, Selangor Malaysia; 208 University of Malaya, Department of Surgery, Faculty of Medicine, Kuala Lumpur, Malaysia; 209 Brigham and Women’s Hospital and Harvard Medical School, Obstetrics and Gynecology Epidemiology Center, Boston, MA USA; 210 Columbia University, Department of Epidemiology, Mailman School of Public Health, New York, NY USA; 211 Magee-Womens Hospital, University of Pittsburgh School of Medicine, Department of Medicine, Pittsburgh, PA USA; 212 McGill University, Program in Cancer Genetics, Departments of Human Genetics and Oncology, Montréal, QC Canada; 213 University of Cambridge, Department of Medical Genetics, Cambridge, UK; 214 Dartmouth College, Geisel School of Medicine, Hanover, NH USA; 215 The Ohio State University, Department of Cancer Biology and Genetics, Columbus, OH USA; 216 Pontificia Universidad Javeriana, Institute of Human Genetics, Bogota, Colombia; 217 University of Oxford, Cancer Epidemiology Unit, Oxford, UK; 218 Beth Israel Deaconess Medical Center, Department of Medical Oncology, Boston, MA USA; 219 University Medical Center Groningen, University Groningen, Department of Genetics, Groningen, The Netherlands; 220 University of Pretoria, Department of Genetics, Arcadia, South Africa; 221 Fundación Pública Galega de Medicina Xenómica, Santiago de Compostela, Spain; 222 Instituto de Investigación Sanitaria de Santiago de Compostela (IDIS), Complejo Hospitalario Universitario de Santiago, SERGAS, Santiago de Compostela, Spain; 223 Vanderbilt University Medical Center, Division of Quantitative Sciences, Department of Obstetrics and Gynecology, Department of Biomedical Sciences, Women’s Health Research, Nashville, TN USA; 224 Duke Cancer Institute, Cancer Control and Population Sciences, Durham, NC USA; 225 Duke University Hospital, Department of Community and Family Medicine, Durham, NC USA; 226 National Institute of Environmental Health Sciences, NIH, Biostatistics and Computational Biology Branch, Research Triangle Park, NC USA; 227 City of Hope, Clinical Cancer Genomics, Duarte, CA USA; 228 Fred Hutchinson Cancer Research Center, Seattle, WA USA; 229 Stanford University School of Medicine, Department of Biomedical Data Science, Stanford, CA USA; 230 Uppsala University, Department of Surgical Sciences, Uppsala, Sweden; 231 University of Malaya, Department of Obstetrics and Gynaecology, University of Malaya Medical Centre, Kuala Lumpur, Malaysia; 232 Hebei Medical University, Fourth Hospital, Department of Molecular Biology, Shijiazhuang, China; 233 National Centre for Scientific Research ‘Demokritos’, Molecular Diagnostics Laboratory, INRASTES, Athens, Greece; 234 Institute of Biochemistry and Experimental Oncology, First Faculty od Medicine, Charles University, Prague, Czech Republic; 235 Women’s Cancer Program at the Samuel Oschin Comprehensive Cancer Institute, Cedars-Sinai Medical Centre, Department of Obstetrics and Gynecology, Los Angeles, CA USA; 236 Royal Pass Road, Tampa, FL USA; 237 University of NSW Sydney, School of Women’s and Children’s Health, Faculty of Medicine, Sydney, NSW Australia; 238 University of NSW Sydney, Adult Cancer Program, Lowy Cancer Research Centre, Sydney, NSW Australia; 239 University of Michigan School of Public Health, Department of Epidemiology, Ann Arbor, MI USA; 240 University of Southern California Norris Comprehensive Cancer Center, Department of Preventive Medicine, Keck School of Medicine, Los Angeles, CA USA; 241 Mayo Clinic, Department of Health Science Research, Division of Epidemiology, Rochester, MN USA; 242 Emory University, Department of Epidemiology, Rollins School of Public Health, Atlanta, GA USA

**Keywords:** Risk factors, Clinical genetics, Genetic markers

Correction to: *European Journal of Human Genetics* (2021) 30:349-362 10.1038/s41431-021-00987-7, published online 14 January 2022

The wrong Supplementary files were originally published with this article; it has now been replaced with the correct files.

## Supplementary information

Table S1

Table S3

Table S4

Table S5

Table S6

