# Supplementary figures and images for: Polygenic risk modeling for prediction of epithelial ovarian cancer risk
Source: Eur J Hum Genet. 2022 Jan 14;30(3):349–62. doi: 10.1038/s41431-021-00987-7 (PMC8904525; doi:10.1038/s41431-021-00987-7)

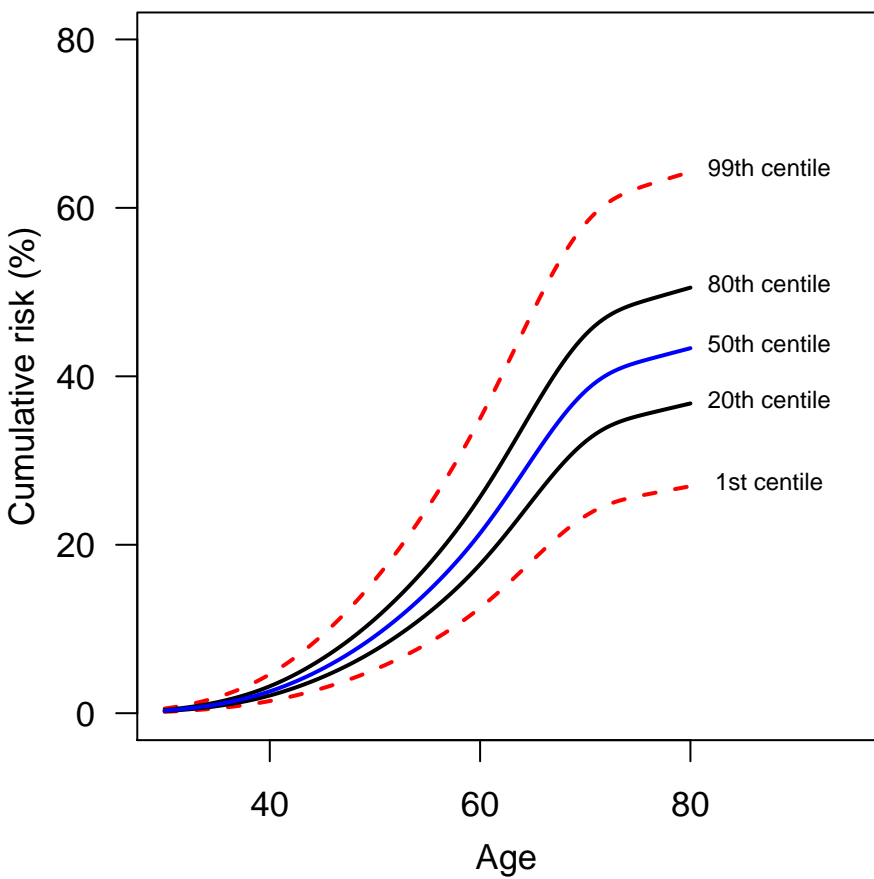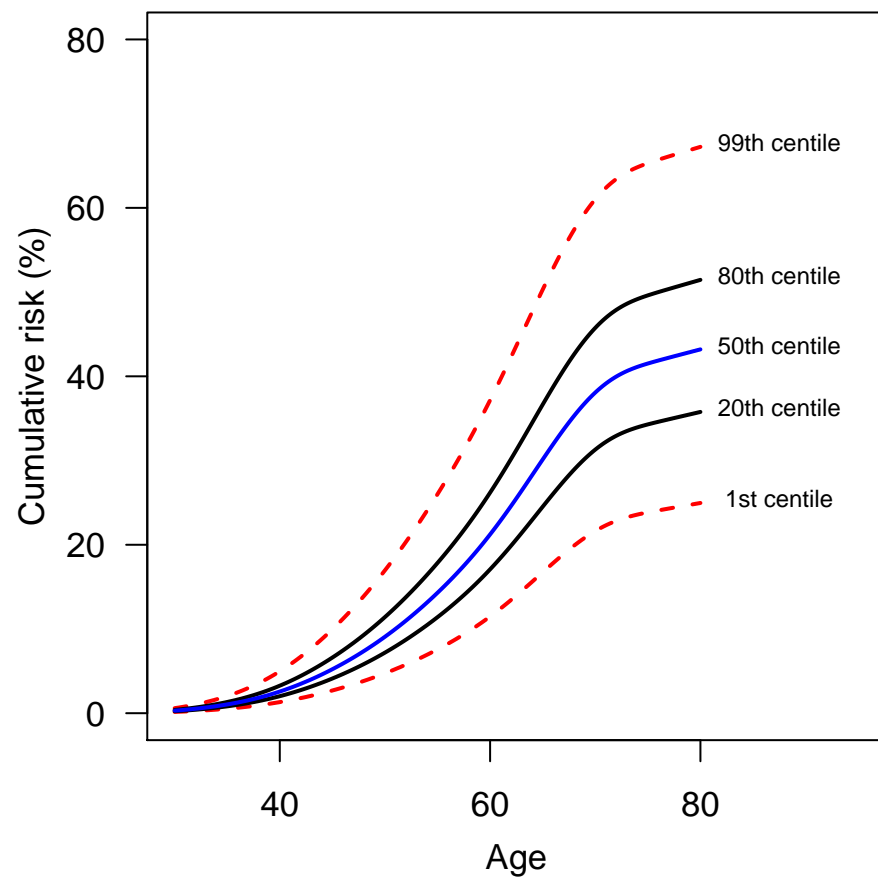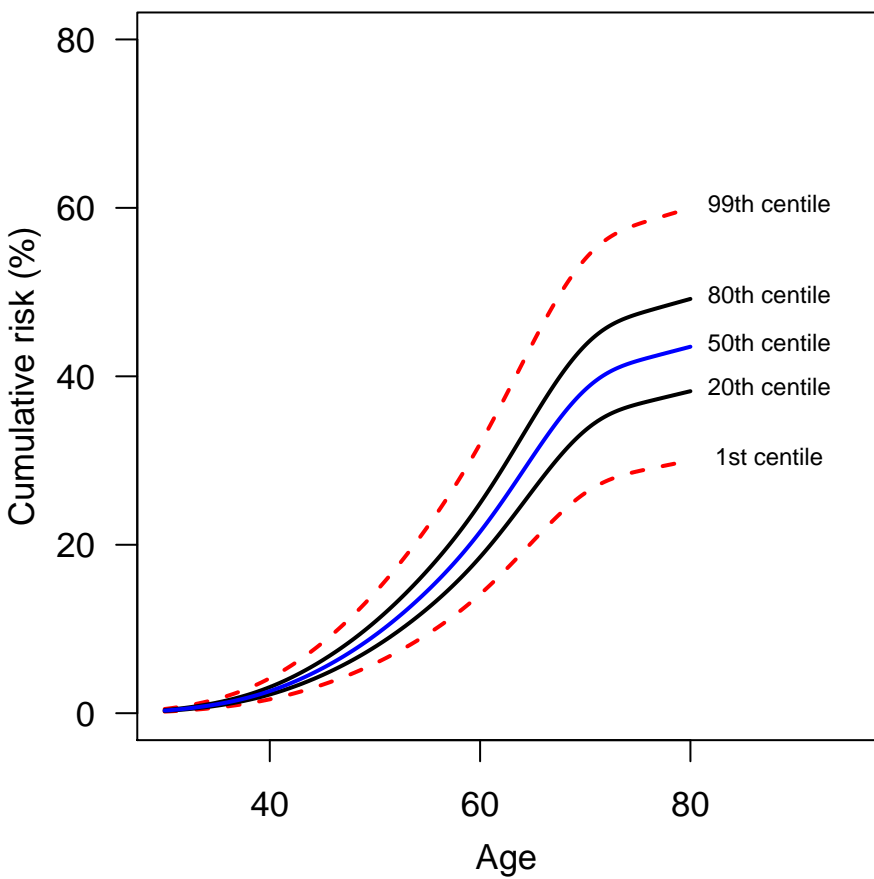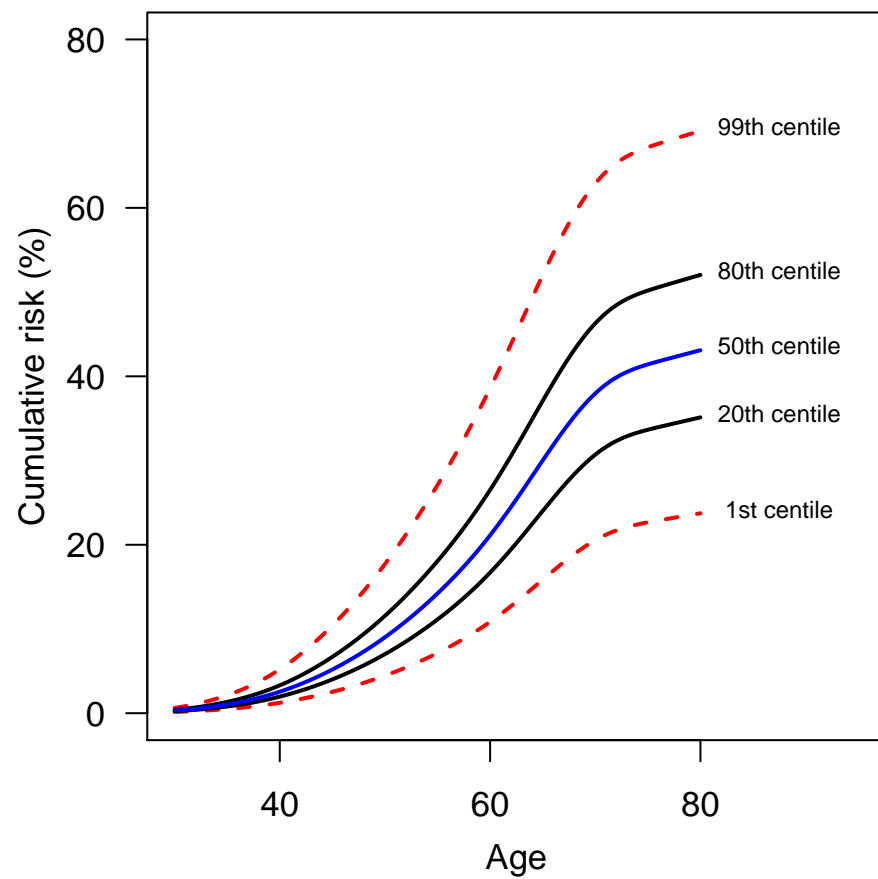

Supplement: Supplementary file 2 — FigureS1: Cumulative risk of ovarian cancer risk in BRCA1 carriers by polygenic risk score percentiles. The lasso (A) and elastic net (B) penalized regression models were applied to individual level g [file 41431_2021_987_MOESM2_ESM.pdf]

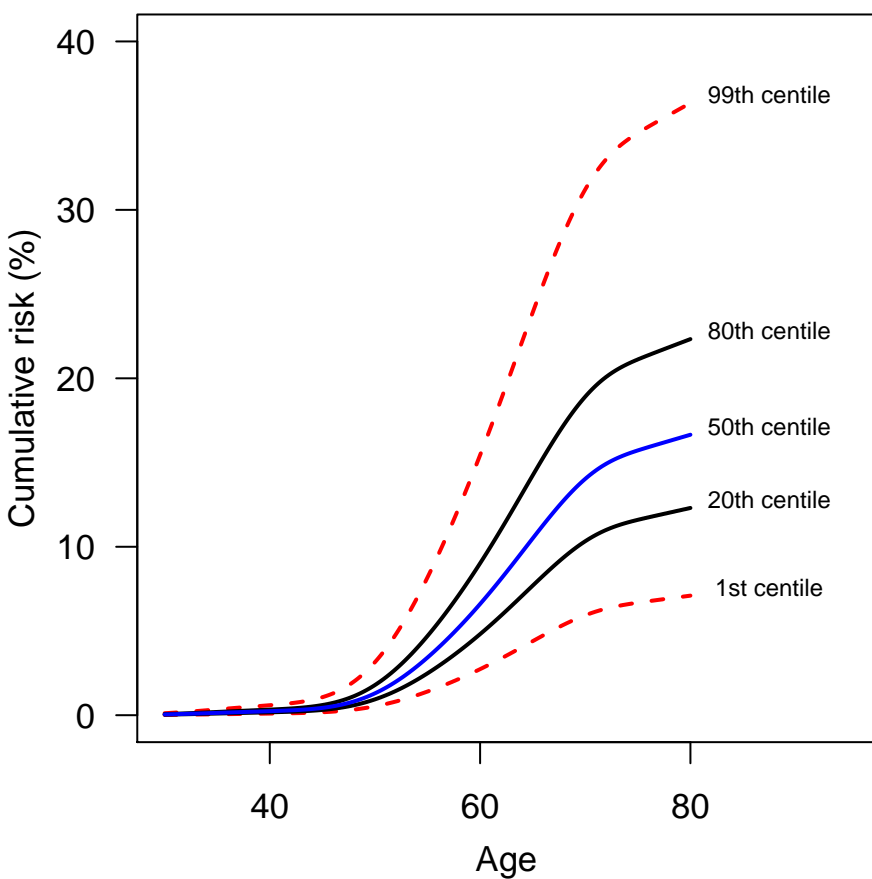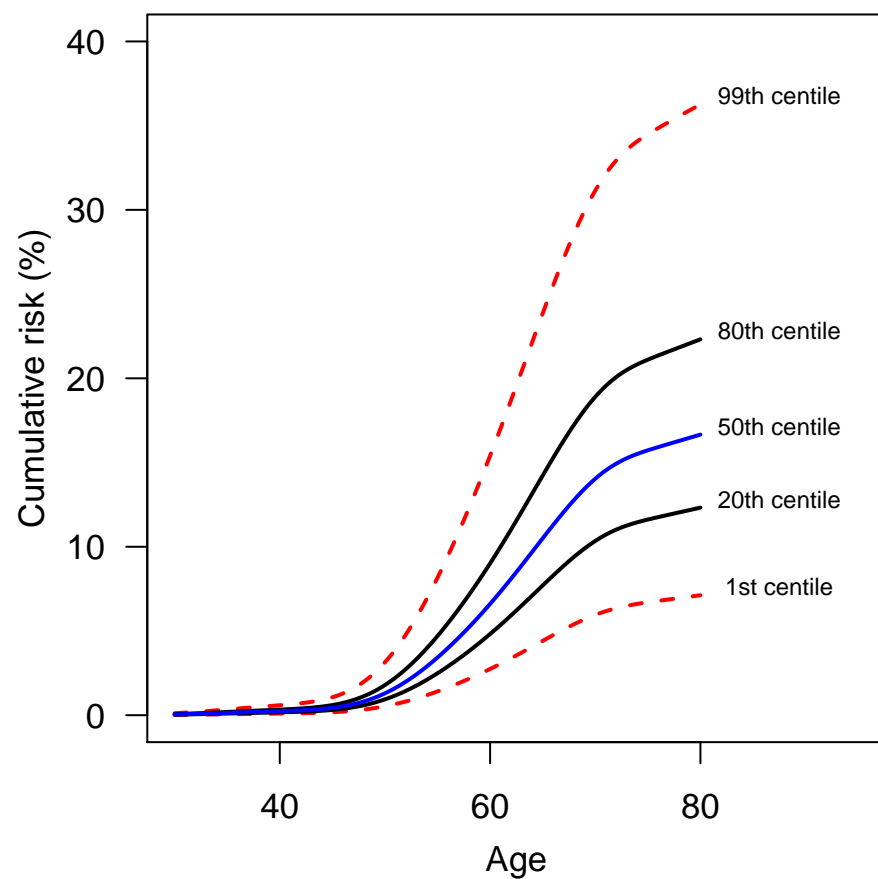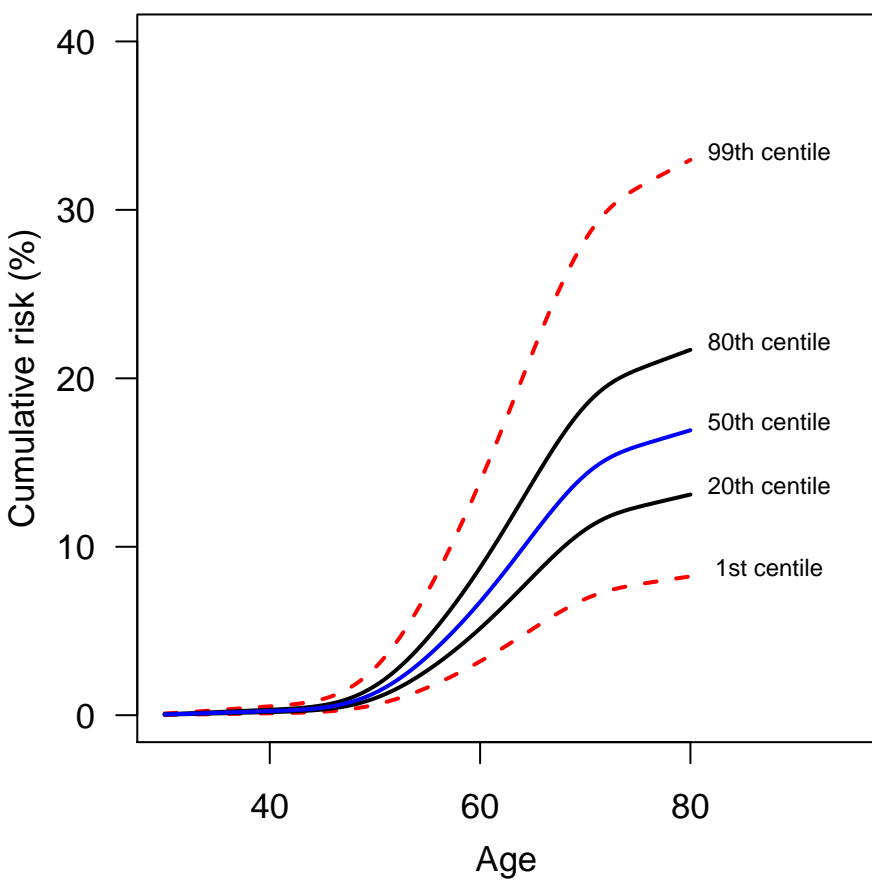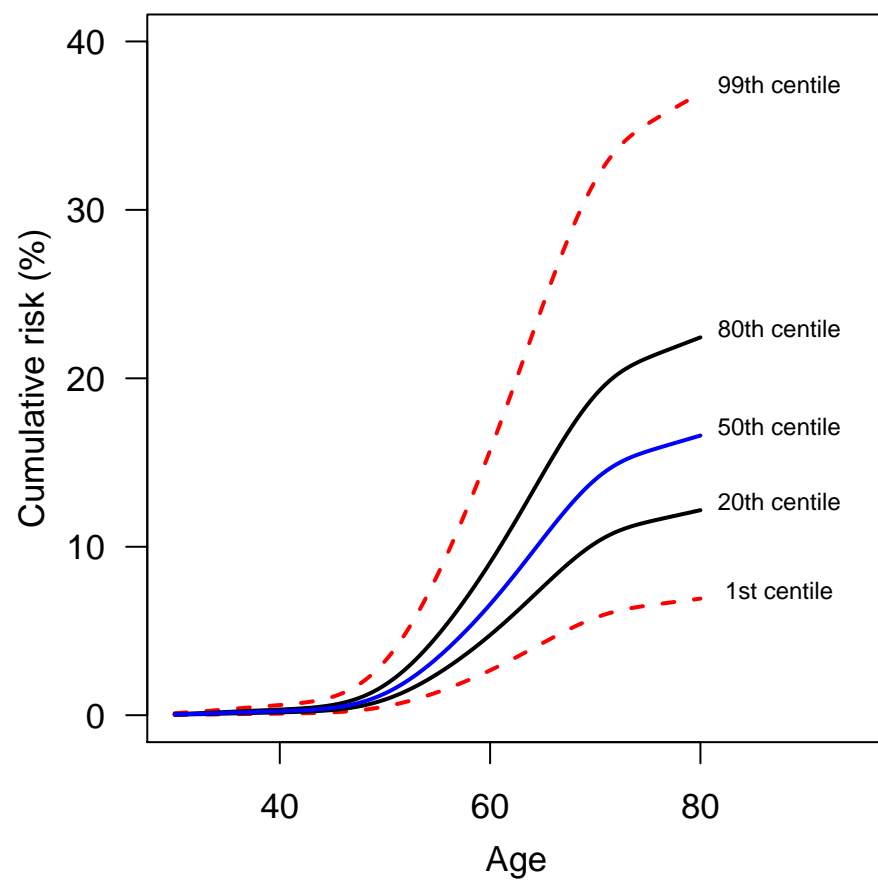

Supplement: Supplementary file 3 — Figure S2:Cumulative risk of ovarian cancer risk in BRCA2 carriers by polygenic risk score percentiles. The lasso (A) and elastic net (B) penalized regression models were applied to individual level g [file 41431_2021_987_MOESM3_ESM.pdf]
